# The Genomic Analysis of Lactic Acidosis and Acidosis Response in Human Cancers

**DOI:** 10.1371/journal.pgen.1000293

**Published:** 2008-12-05

**Authors:** Julia Ling-Yu Chen, Joseph E. Lucas, Thies Schroeder, Seiichi Mori, Jianli Wu, Joseph Nevins, Mark Dewhirst, Mike West, Jen-Tsan Chi

**Affiliations:** 1 Institute for Genome Sciences and Policy, Duke University, Durham, North Carolina, United States of America; 2 Department of Molecular Genetics and Microbiology, Duke University, Durham, North Carolina, United States of America; 3 Department of Statistical Science, Duke University, Durham, North Carolina, United States of America; 4 Department of Radiation Oncology, Duke University, Durham, North Carolina, United States of America; The University of Queensland, Australia

## Abstract

The tumor microenvironment has a significant impact on tumor development. Two important determinants in this environment are hypoxia and lactic acidosis. Although lactic acidosis has long been recognized as an important factor in cancer, relatively little is known about how cells respond to lactic acidosis and how that response relates to cancer phenotypes. We develop genome-scale gene expression studies to dissect transcriptional responses of primary human mammary epithelial cells to lactic acidosis and hypoxia *in vitro* and to explore how they are linked to clinical tumor phenotypes *in vivo*. The resulting experimental signatures of responses to lactic acidosis and hypoxia are evaluated in a heterogeneous set of breast cancer datasets. A strong lactic acidosis response signature identifies a subgroup of low-risk breast cancer patients having distinct metabolic profiles suggestive of a preference for aerobic respiration. The association of lactic acidosis response with good survival outcomes may relate to the role of lactic acidosis in directing energy generation toward aerobic respiration and utilization of other energy sources via inhibition of glycolysis. This “inhibition of glycolysis” phenotype in tumors is likely caused by the repression of glycolysis gene expression and Akt inhibition. Our study presents a genomic evaluation of the prognostic information of a lactic acidosis response independent of the hypoxic response. Our results identify causal roles of lactic acidosis in metabolic reprogramming, and the direct functional consequence of lactic acidosis pathway activity on cellular responses and tumor development. The study also demonstrates the utility of genomic analysis that maps expression-based findings from *in vitro* experiments to human samples to assess links to *in vivo* clinical phenotypes.

## Author Summary

It is well recognized that tumor microenvironments play an important role in modulating tumor progression in human cancers. Although previous studies have highlighted the importance of hypoxia, there is limited knowledge on the effects of other components in tumor microenvironments. Therefore, we use gene expression to compare and analyze how cells respond to lactate, acidity, and hypoxia, as well as how these responses can be utilized to predict the clinical outcomes of patients with breast cancers. We uncover an unexpected association with better clinical outcome of the strong lactic acidosis and acidosis response in breast cancers as a result of their abilities to inhibit glycolysis and favor oxidative phosphorylation for energy generation. This effect is caused by not only the repression of the gene expression of glycolysis genes but also the inhibition of Akt activation of cells exposed to lactic acidosis and acidosis. In conclusion, we propose that lactic acidosis and acidosis to be considered as independent prognostic factors for human cancers.

## Introduction

The tumor microenvironment is characterized by oxygen depletion (hypoxia), high lactate and extracellular acidosis (lactic acidosis) as well as glucose and energy deprivation [1]. These changes are largely caused by a combination of poor tissue perfusion, abnormal tumor vasculature, uncontrolled proliferation and dysregulated energy metabolism. These microenvironmental features vary widely in different tumors, reflecting heterogeneity in the metabolic status of individual tumors. They can also trigger phenotypic changes in cancer cells and directly modulate biological properties and clinical phenotypes. Although our understanding of hypoxia has advanced tremendously in recent years, relatively little is known about the role of other microenvironmental stresses, especially lactic acidosis and glucose starvation.

The accumulation of lactic acid in solid tumors is often thought to be caused by tumor hypoxia – a by-product of glycolysis as the tumor cells shift to an anaerobic mode of energy production under hypoxia or due to the altered metabolic profiles of cancer cells. In spite of this apparent mechanistic link, these two factors exhibit significant disparities in their spatial and temporal distribution in tumors [2],[3]. This may be due to the fact that some tumors exhibit a predisposition toward glycolysis even in the presence of oxygen, a phenomenon that is referred to as aerobic glycolysis or the Warburg effect [4]. Some tumors may also possess greater capacity to pump protons out to the extracellular space to create a reversed pH gradient – acidic extracellular pH (pHe) and alkaline intracellular pH (pHi) through higher expression of proton transporters [5]. Additionally, lactic acid can accumulate in poorly perfused tissue due to inefficient removal. Thus, it is important to consider these two factors separately in understanding their distinct contributions to tumor phenotypes.

Research on tumor microenvironments relies on our ability to manipulate *in vitro* conditions of mammalian cell growth to re-create the relevant stresses experienced *in vivo*. This offers a means to explore the roles of individual environmental factors on cellular behavior and phenotype. Genetic and pharmacological manipulation can then be applied to explore the molecular mechanisms and genetic circuitry underlying cellular responses. Such studies typically observe *in vitro* cellular behavior and make inferences about contributions to tumor progression *in vivo*. For example, hypoxia, when applied *in vitro*, has been shown to promote angiogenesis, cellular migration and energy consumption, thus providing the potential mechanisms for its association with poor clinical outcome [6],[7]. Similarly, lactic acidosis, when applied to cultured cells, has been shown to trigger calcium signaling [8], gene expression of angiogensis (e.g. VEGF, IL8) [9],[10],[11], HIF1α stabilization [12], cell death [13] and affect gene expression [14],[15],[16]. Recent studies also use genomic analysis to identify the cellular response to acidosis and high lactate [14],[16]. These results are summarized in several nice reviews [17],[18],[19]. However, inference using these observations from *in vitro* perturbations to *in vivo* cancer phenotypes is frequently challenging and indirect. Gene expression microarray signatures have provided a solution to this gap since they afford an opportunity to develop a surrogate phenotype of the *in vitro* state which can then be assessed in the *in vivo* state. This approach generates gene expression signatures from perturbations in cultured cells *in vitro* to represent a defined biological process [20],[21],[22], which in turn can serve as a common phenotype to recognize similar molecular features in human cancer samples *in vivo*. Using this approach, we have previously shown that the wound healing, hypoxia responses and various oncogenic mutations can play important roles in tumor progression [20],[21],[23]. Our current study applies this strategy to lactic acidosis, aiming to elucidate the casual roles of lactic acidosis at a molecular level and evaluate the prognostic implications. The analysis reported in this manuscript investigates these molecular mechanisms through integrative genomic analysis of lactic acidosis responses from both *in vitro* culture cells and *in vivo* human cancers.

## Results

### Dissection of Gene Expression Responses of Hypoxia and Lactic Acidosis

**Figure 1:**
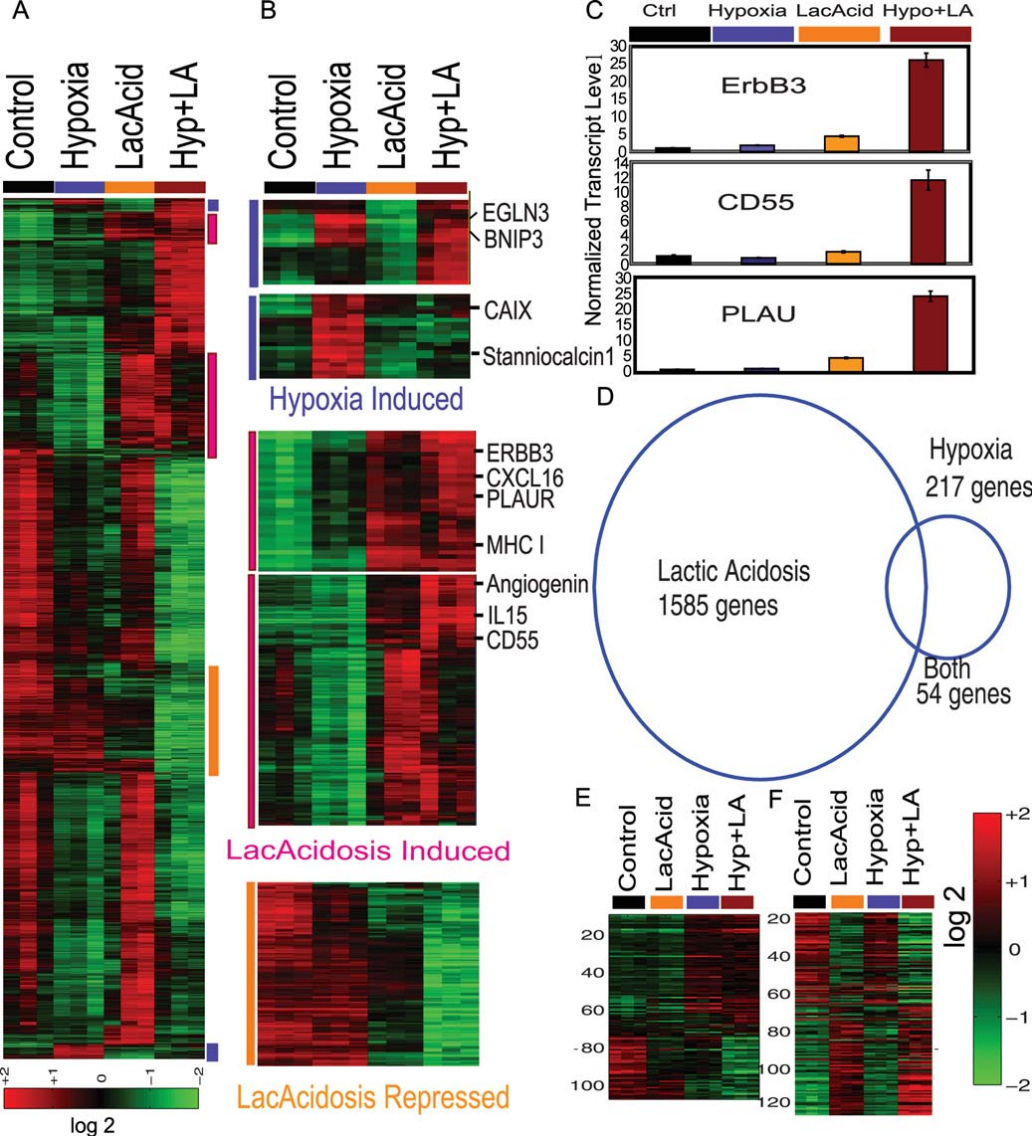
Overview of the cellular responses to hypoxia and lactic acidosis. (A,B) The gene expression response of HEMC is shown when exposed to control, hypoxia, lactic acidosis and combined hypoxia and lactic acidosis conditions. 4722 genes with expression variations of at least 1.75 fold in two samples were selected and hierarchically clustered. Genes induced by hypoxia (vertical blue bar), lactic acidosis (pink), and repressed by lactic acidosis (orange) are marked and further expanded in (B). (C) The expression of three lactic acidosis-induced genes ERBB3, CD55 and PLAUR normalized by actin-beta were confirmed by real time RT-PCR. Similar results were observed when normalized by another control gene, B2M (D) Venn diagram showing the number of genes changed by lactic acidosis (1585 genes), hypoxia (217 genes) and overlap (54 genes) for whom the probability of an expression change exceeded 0.99. (E, F) The expression of genes comprising hypoxia (E) and genes comprising lactic acidosis (F) gene signatures was shown in respective heat maps in all four indicated conditions.

To characterize the gene expression program generated in response to lactic acidosis, hypoxia, and combined lactic acidosis and hypoxia, we made use of human mammary epithelial cells (HMEC) brought to replicative arrest by growth factor/serum withdrawal for 24 hours. Since the HMEC represent normal epithelial cells with intact signaling components, the response elicited in HMECs is likely to reflect the cellular response not biased by genetic mutations present in cancer cell lines. We exposed HMECs to four different culture environments for 24 hours in triplicate samples: 1) control – ambient oxygen level (∼21%O_2_) with neutral pH; 2) lactic acidosis – 25 mM lactic acidosis with pH 6.7; 3) hypoxia (2% O_2_) with neutral pH; 4) combined lactic acidosis and hypoxia. We did not find a significant change in media pH at the end of the 24 hour culture. The gene expression of these HMEC samples were interrogated with Affymetrix GeneChip U133 plus 2.0 arrays to measure the expression of more than 54,000 probe sets and at least 47,000 transcripts and variants. Gene expression profiles of cellular responses to hypoxia, lactic acidosis and combined stresses were first normalized by RMA, mean centered and filtered with the criteria of at least 2 (out of 3 samples in each experimental condition) observations with at least 1.75 fold changes to select 4722 probes sets. A clustering analysis on these genes revealed that hypoxia and lactic acidosis induced distinct sets of genes (Figure 1A). The hypoxia induced gene clusters included CA9, stanniocalcin1, EGLN3, BNIP3 and many of the genes seen in our prior studies with spotted cDNA arrays [21] (Figure 1B). Interestingly, induction of some hypoxia-induced genes (e.g. CA9, Stanniocalcin1) was abolished by simultaneous lactic acidosis (Figure 1B), consistent with previous studies [24],[25].

Compared with hypoxia, the lactic acidosis response is more dramatic with alterations of many more genes. Genes induced by lactic acidosis included: PLAUR, ERBB3, CD55, interleukin 15, CXCL16, angiogenin and MHC class I genes (Figure 1B). For a subset of these lactic acidosis-induced genes, the degree of induction was further enhanced by the combined stresses of lactic acidosis and hypoxia. Among genes repressed by lactic acidosis, many are involved in cell cycle, cell proliferation and glucose metabolism (Figure 1B). We further confirmed the induction of ERBB3, CD55, and PLAUR in response to lactic acidosis, and to the combination of hypoxia with lactic acidosis, via real-time PCR (Figure 1C).

A supervised analysis of the full set of data on all 47,000 probe sets was performed using Bayesian multivariate regression analysis (BFRM) that has been utilized in a number of prior studies [26],[27] (Text S1). This analysis includes the ability to use housekeeping gene information on each chip in order to automatically correct for gene-sample specific assay artifacts. The multivariate analysis computes, among other things, gene-specific probabilities of expression changes resulting from lactic acidosis or hypoxia stress (Table S1). At a threshold of probability of expression change of 0.99 (Bayesian significance of 1%) we find 217 genes whose expression is significantly altered by hypoxia and 1585 genes by lactic acidosis; only 54 genes are affected by both individual treatments (Figure 1D). Cellular responses to lactic acidosis are more dramatic and wide-spread than the responses to hypoxia, and involve substantially distinct gene sets (Figure 1B, D).

To survey the molecular pathways triggered by lactic acidosis and hypoxia, we analyzed the Gene Ontology (GO) enrichment in the genes induced and repressed by hypoxia and lactic acidosis using GATHER [28]. Among the genes induced by lactic acidosis, we found enrichment for G-protein coupled receptor signaling, antigen processing and presentation, and cellular catabolism (Table S2). The top GO terms repressed by lactic acidosis were genes involved in cell cycle, RNA metabolism and RNA processing (Table S2). On the other hand, the top GO terms enriched in the hypoxia-induced genes included hexose metabolism, glycolysis, glucose metabolism and glucose catabolism (Table S3); while the GO terms enriched in the hypoxia-repressed genes included cell cycles and RNA metabolism (Table S3). Furthermore, we compared the GO terms enriched when cells are exposed to hypoxia and lactic acidosis together (Table S4).

Previous studies have shown that lowering the extracellular pH from 7.4 to ∼6.7 will lead to a slight lowering of intracellular pH (pHi) from 7.4 to 6.9–7.0, which is likely to be mediated by monocarboxylate transporter (MCT) proteins [29],[30]. Given the importance of MCT family proteins in the regulation of cellular response to lactic acidosis, we analyzed this family of proteins under both hypoxia and lactic acidosis (Table S5). We found that MCT-4 was induced significantly under hypoxia and repressed under lactic acidosis. The induction of MCT-4 by hypoxia has been previously reported [31].

To further assess the extent to which the hypoxia and lactic acidosis response overlaps, we used binary logistic regression to estimate the probability of activation for the overall hypoxia and lactic acidosis pathways revealed in gene expression under individual conditions. We first used the control vs. hypoxia (for hypoxia probability) and control vs. lactic acidosis (for lactic acidosis probability) groups to provide the training sets to generate respectively hypoxia (Figure 1E) and lactic acidosis (Figure 1F) gene signatures. These signatures were then used to estimate their probability in the remaining two groups of samples in the same experiment. We found that the there is only marginal hypoxia pathway activation evident in lactic acidosis samples compared to controls (0.158 (control) vs. 0.286 (lactic acidosis), p = 0.14, Figure S1A). A similar analysis on the lactic acidosis pathway in hypoxia response samples indicated slightly elevated levels of lactic acidosis relative controls (0.362 (hypoxia) vs. 0.231 (control), p = 0.005, Figure S1B). We also used the BFRM analysis to identify genes which are altered only in the presence of both hypoxia and lactic acidosis. At a threshold of 99% probability, we found 127 induced genes and 320 repressed genes in the presence of both hypoxia and lactic acidosis (Figure S2A, B). Among the GO terms enriched in the induced genes were several transcription factors involved in regulating transcriptional activities (ATF3, YY1, CPEB2/3, SAP18, SREBF2, Figure S2A). Among the GO terms enriched in the repressed genes were genes encoding proteins involved in apoptosis process (FADD, CASP6, PDCD6, ERCC3, Figure S2B). The down-regulation of these pro-apoptotic genes may be important in maintaining the cellular survival during the simultaneous presence of both environmental stresses.

### Dissection of Gene Expression Responses of Lactosis and Acidosis

**Figure 2:**
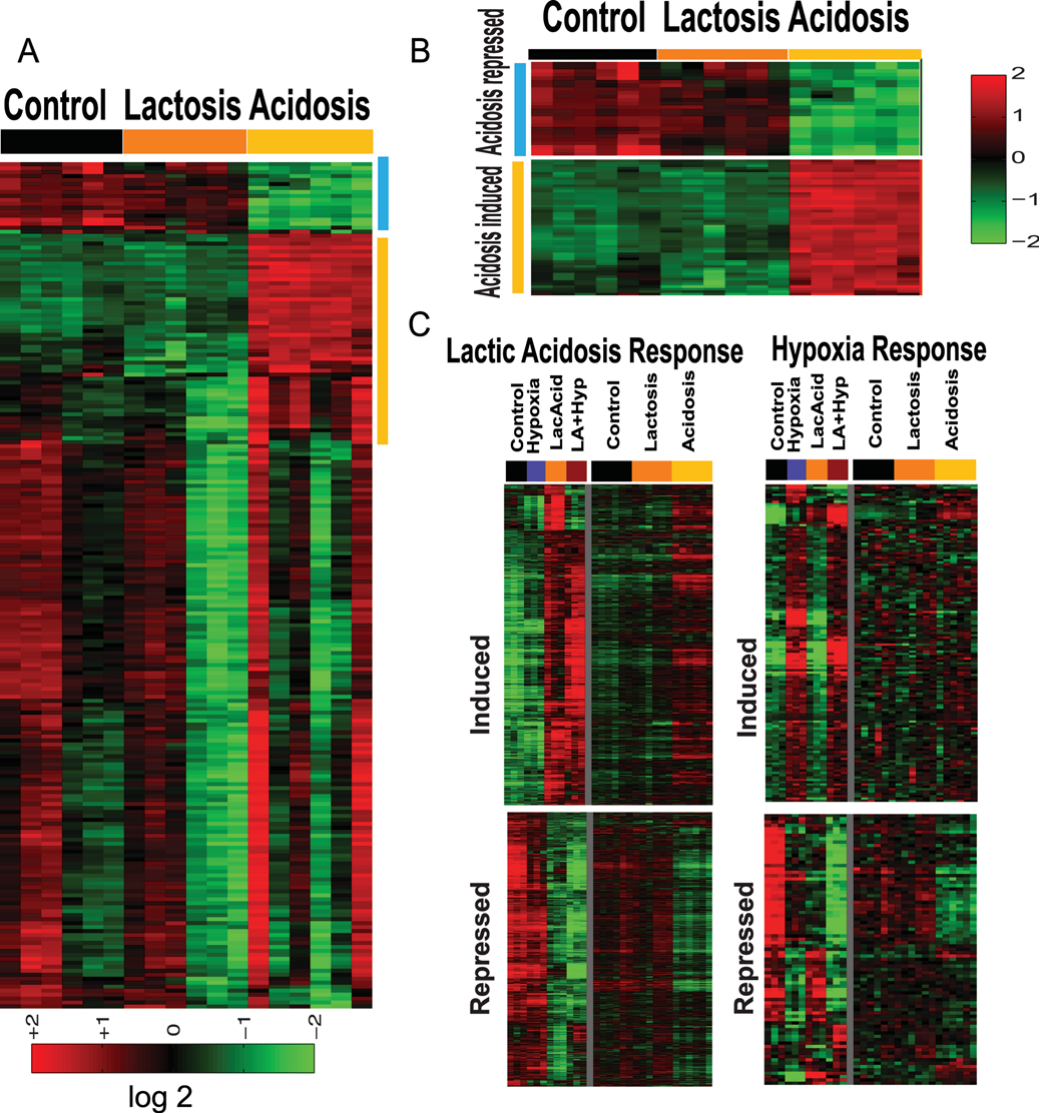
The cellular responses to lactosis and acidosis. (A) HMECs were exposed to three indicated environments: control, lactosis, and acidosis. 213 genes with expression varied from the mean at least 1.75 fold in 4 samples were selected and hierarchically clustered. A cluster of genes strongly induced by acidosis is shown (yellow vertical bar). (B) The expression of genes comprising acidosis gene signatures under indicated conditions was shown in heat maps. The expression of genes selected by statistical analysis (see Text S1) for whom the probability of an expression change under lactic acidosis (C) or hypoxia (D) exceeds 0.99 were shown under hypoxia/lactic acidosis or lactosis/acidosis. Most lactic acidosis-induced/repressed genes are also induced/repressed by acidosis but not lactosis.

High lactate (lactosis) and low pH (acidosis) often co-exist in tumor lactic acidosis, but these two factors are not necessarily present simultaneously. To determine the respective contributions of lactosis and acidosis in the lactic acidosis response, we created culture conditions to separate the lactic acidosis condition (pH 6.7 created by 25 mM lactic acid) into lactosis (25 mM sodium lactate with neutral pH) and acidosis (pH 6.7 created by HCl) conditions. We analyzed the transcriptional responses of HMECs under lactosis and acidosis via microarrays. Gene expression profiles of the cellular responses to lactosis and acidosis were normalized by RMA, mean centered and filtered with the criteria of at least 4 (out of 6 sample in each experimental condition) observations with at least 1.75 fold change. 213 probes were identified and clustered in Figure 2A. Acidosis induced a much more dramatic change in gene expression than lactosis. Acidosis-induced genes include many of the genes also induced by lactic acidosis in our previous analysis (Figure 1B). We also confirmed with realtime RT-PCR of the induction of ERBB3 and SOD2 in response to acidosis (Figure S3). The acidosis gene signatures were determined by comparing the 6 control vs. 6 acidosis samples (Figure 2B). Most genes in the acidosis gene signature were not altered under lactosis (Figure 2B). To assess the relative contributions of acidosis and lactosis to the lactic acidosis response, we compared the expression level of genes changed in the three groups. We find a high concordance between lactic acidosis and acidosis responses, with similar sets of genes induced and repressed under these two stresses (Figure 2C, Table S6). In contrast, this concordance is not present in other comparisons between lactosis vs. lactic acidosis (Figure 2C), hypoxia vs. lactic acidosis or hypoxia vs. acidosis treatment (Figure 2D). This suggests that lactic acidosis (created by lactic acid) and acidosis (created by HCl) trigger similar genetic responses, distinct from genetic responses to lactosis and hypoxia.

### Genomic Analysis of Hypoxia and Lactic Acidosis Response in Human Cancers

**Figure 3:**
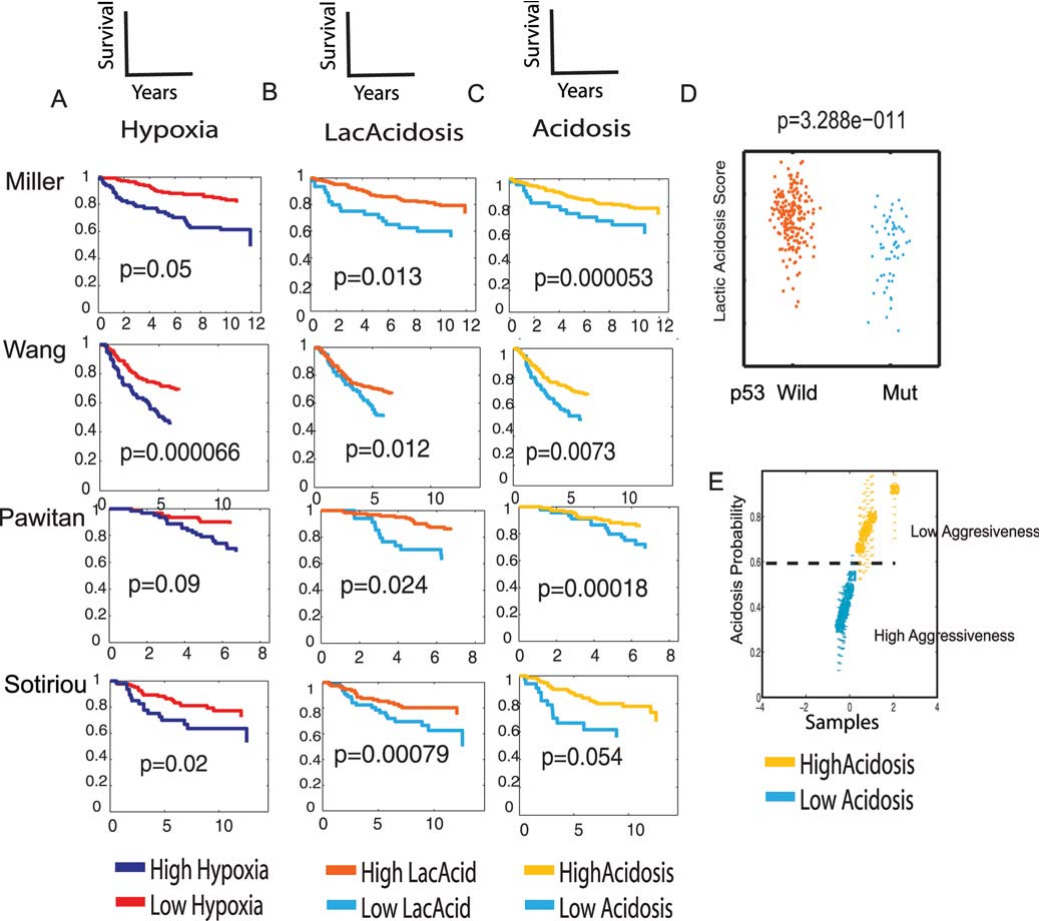
The prognostic significance of gene signatures reflecting hypoxia, lactic acidosis and acidosis response in human breast cancers. The gene signatures in hypoxia (A), lactic acidosis (B), and acidosis (C) response were assessed in the four indicated breast cancer expression datasets. The tumors stratified by the degrees of these responses were used to generate the Kaplan-Meier survival curves for the clinical outcomes exhibiting high and low indicated responses are shown. (D) In the Miller dataset, the lactic acidosis response score is significantly higher among the tumors with wild type p53 than mutant p53 (p = 3.288×10^−11^). (E) Acidosis response were also estimated for a group of breast cancer cell lines with different metastatic abilities and found to be negatively correlated with tumor aggressiveness determined in xenografted mice [36].

We previously showed that the hypoxia response elicited in cultured epithelial cells provides a molecular gauge of hypoxia response for cancerous human tissues *in vivo* and predicts poor clinical outcome [21]. This involves projecting the *in vitro* gene signature of hypoxia “response” or “pathway activity” into numerical scores on each tumor sample in the *in vivo* expression data to assess the corresponding predicted levels of hypoxia pathway activity in each tumor. Similar approaches have been used in other studies using *in vitro* generated signatures to infer *in vivo* responses or pathway activities in tumors [20],[21],[23],[32],[33]. The hypoxia response signature was evaluated in a number of breast data sets via a weighted average of the signature gene set based principal components analysis (full details in Statistics Supplement). Analysis of a Cox survival model indicates patients with tumors showing higher levels of hypoxia pathway activity had poorer clinical outcomes (Figure 3A, Miller), consistent with our previous studies [21]. (Reported p-values are from the variable relevant to the figure, pathway activity score for the indicated pathway, used in a Cox survival model.) Completely concordant results were also observed in three other breast cancer expression studies with different stages of diseases (Figure 3A). These datasets include a study of 286 lymph node negative early breast cancers from NKI (Wang), and two studies of invasive breast carcinomas (Sotiriou, Pawitan) [34],[35]. Similar trends are present but not statistically significant in one smaller study of 82 breast cancers with information on distant metastasis [36] (data not shown).

Given the perceived relation of lactic acidosis with hypoxia in tumors, we evaluated the prognostic value of the lactic acidosis signature using the same statistical approach. We found that tumors with high lactic acidosis response signatures have significantly improved overall survival, in contrast to that for hypoxia response signature. This association with favorable clinical outcomes is consistent across all four breast cancer datasets with their different target populations and stages of cancers (Figure 3B). Given the similarity of lactic acidosis and acidosis signatures, we also assessed the acidosis gene signature alone and confirmed that tumors with high acidosis response signature activity exhibited better clinical outcome and survival (Figure 3C). In the Miller dataset with information on p53 status of individual tumors, we also found a strong association between lactic acidosis and p53 status: the estimated “lactic acidosis pathway activity” based on gene expression is significantly higher in wild type p53 tumors than in those with p53 mutations (p = 3.288×10^−11^) (Figure 3D).

Among the breast cancer patients included in the Sotiriou dataset, 64 patients were treated with tamoxifen while the remaining 125 patients were untreated. Both hypoxia and lactic acidosis pathway signatures are predictive of poor and favorable outcomes respectively in the patients treated with tamoxifen (Figure S4A, B), but less so among the untreated patients in this cohort (Figure S4C, D).

To understand whether the prognostic value of lactic acidosis is evident in a cell autonomous manner, we tested the prognostic value of the lactic acidosis signature in various breast cancer cell lines grown in a controlled culture milieu. We used binary logistic regression to determine the probability of acidosis response in each of a set of breast cancer cell lines that vary in their potential for distant metastasis [36]. The acidosis signature probability varied greatly among different cell lines; cell lines with high acidosis signature demonstrated low levels of aggression in the xenograft model when compared to those with low acidosis signatures (Figure 3E) [36]. This indicates that the prognostic information contained in the acidosis signature reflects the intrinsic phenotypes of these cancer cell lines.

We also tested the prognostic value of gene signatures reflecting lactic acidosis response and hypoxia response in a multivariate survival analysis using the Sotiriou data set. When both gene signatures are included in the Cox survival model on all samples for which we have survival, ER status, tumor size, and data on node involvement, the p-values for lactic acidosis and hypoxia are 0.0379 and 0.0069 respectively. When we include these two pathway variables along with clinical variables indicating ER status, tumor size >2 cm and node involvement, the p-values are .07, .008, .85, .0032 and .77 respectively. Dropping ER status and node involvement (poor predictors for this dataset) gives p-values of .06, .008, and .002.

We further evaluated parametric Weibull survival models involving different combinations of clinical variables and expression signatures. Figure S5 presents some summary survival curves comparing “high” versus “low” risk groups based on samples “below” versus “above” the median predicted survival time. This analysis shows that survival model involving lactic acidosis, hypoxia, and node size perform almost identically to models involving all variables (Figure S5A, B). Further, models involving just clinical variables perform worse than those that include both clinical and signatures. Thus the clinical and pathway variables provide synergistic value in predicting outcomes in breast cancer patients.

### Relationship between the Hypoxia and Lactic Acidosis Responses in Human Breast Cancers

**Figure 4:**
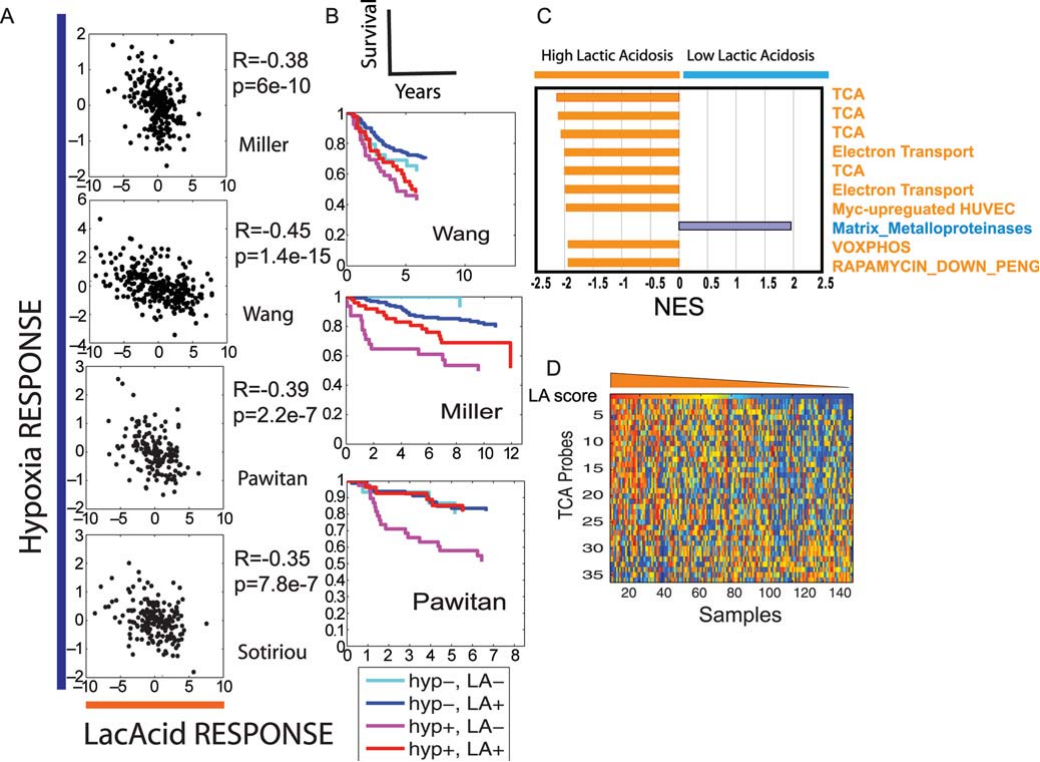
The exploration of lactic acidosis response in breast cancers. (A) Scatter plots showing the relationship between the probability of hypoxia response (Y-axis) and lactic acidosis response (X-axis). Each point in the scatter plots represents a single tumor from the indicated breast cancer data sets. The overall correlation (R) and probability (p) between hypoxia and lactic acidosis signatures across all samples is shown for the indicated data set. (B) The tumors in the indicated breast cancer data set are separated into four groups based on hypoxia and lactic acidosis responses. Kaplan-Meier curves for the clinical outcomes of these four groups of tumors are shown with indicated colors. (C) The top ten genesets based on normalized enrichment score (NES) from GSEA analysis for the difference in pathway composition between the tumors with high vs. low lactic acidosis responses in the Pawitan data. (D) The breast samples in the Pawitan data was arranged from left to right by descending lactic acidosis score (top row). The expression of genes in TCA cycles in these samples is shown in heat map (orange means higher expression whereas blue means lower expression) together with lactic acidosis score.

Since lactic acid production and accumulation in solid tumors is likely to relate to the shift to glycolysis under hypoxia, a high degree of correlation is expected between hypoxia and lactic acid in solid tumors. What is the relationship between the degree of hypoxia and lactic acidosis responses in the same tumors? With our quantitative, probabilistic assessment of the pathway activities based on tumor gene expression, we can directly investigate the relationship between these two factors in individual tumors. Unexpectedly, the lactic acidosis response score correlated significantly in a negative fashion with the hypoxia score in all four expression studies, with R ranging from −0.35 to −0.45 and *p* values from 2.2E-7 to1.4E-15 (Figure 4A). Although this negative correlation between the lactic acidosis and hypoxia responses is somewhat unexpected, it is consistent with the findings that a strong lactic acidosis response predicts favorable prognosis, (Figure 3B) while strong hypoxia response predicts poor prognosis (Figure 3A). To test the potential for synergistic value when both gene signatures are combined in patient stratification, we found the ability to predict survival is significantly improved when we combine the two signatures in breast cancers survival prediction (Figure 4B).

### Lactic Acidosis Response Favors Aerobic Respiration as Means of Energy Generation

To understand the unexpected association between lactic acidosis response and favorable clinical outcomes, we compared the pathway composition between tumors with high vs. low lactic acidosis responses in all four expression datasets using Gene Set Enrichment Analysis (GSEA) [37]. GSEA uses a Kolmogorov-Smirnov statistic to determine whether specific biological processes (represented by gene sets) are significantly enriched in a subset of breast tumors with strong lactic acidosis response [37]. Among the top gene sets enriched in tumors with strong lactic acidosis responses, two biological processes are prominent: aerobic/mitochondria respiration (e.g. Kreb cycles, electric transportation and oxidative phophorylation) and the metabolism of fatty acids and amino acids (Figure 4C, Table S7). For example, the 46 genes in the TCA cycles compiled by KEGG pathway database are highly enriched in the breast cancers with strong lactic acidosis pathways in the Pawitan study (Figure S6). Several gene sets representing aerobic respiration from other sources also show significant enrichment. When all the samples in the Pawitan study were ranked based on their lactic acidosis score, the tumors with high lactic acidosis response tend to have higher expression level of genes in TCA cycles (Figure 4D). In contrast, tumors with high hypoxia response tend to have lower expression level of genes in the TCA cycle (Figure S7). Thus a high lactic acidosis response identifies a group of breast cancers enriched in the use of aerobic respiration. It is interesting to note that several gene sets representing amino acid and fatty acid metabolism are also enriched in these tumors (Table S7), reflecting the distinct metabolic profiles and mode of energy generation in tumors with high lactic acidosis responses.

There are two major pathways for ATP-generation in mammalian cells – glycolysis or aerobic respiration. One of the fundamental properties of cancer cells is their preferential utilization of glycolysis over aerobic respiration to produce ATP. The glycolytic phenotype of cancer cells is thought to offer selective advantages since the disruption of glycolysis phenotype (e.g. silencing of LDH-A) results in stimulation of mitochondrial respiration and significantly compromises their tumorigenicity and the proliferation under hypoxia [38]. Our GSEA result suggests the lactic acidosis gene signature can identify a subgroup of tumors with a higher level of aerobic respiration and more favorable clinical outcomes. This association between high lactic acidosis activity and strong aerobic respiration is also consistent with its links to wild type p53 (Figure 3D) since p53 has been shown to redirect the metabolic pathways toward aerobic respiration [39],[40].

**Figure 5:**
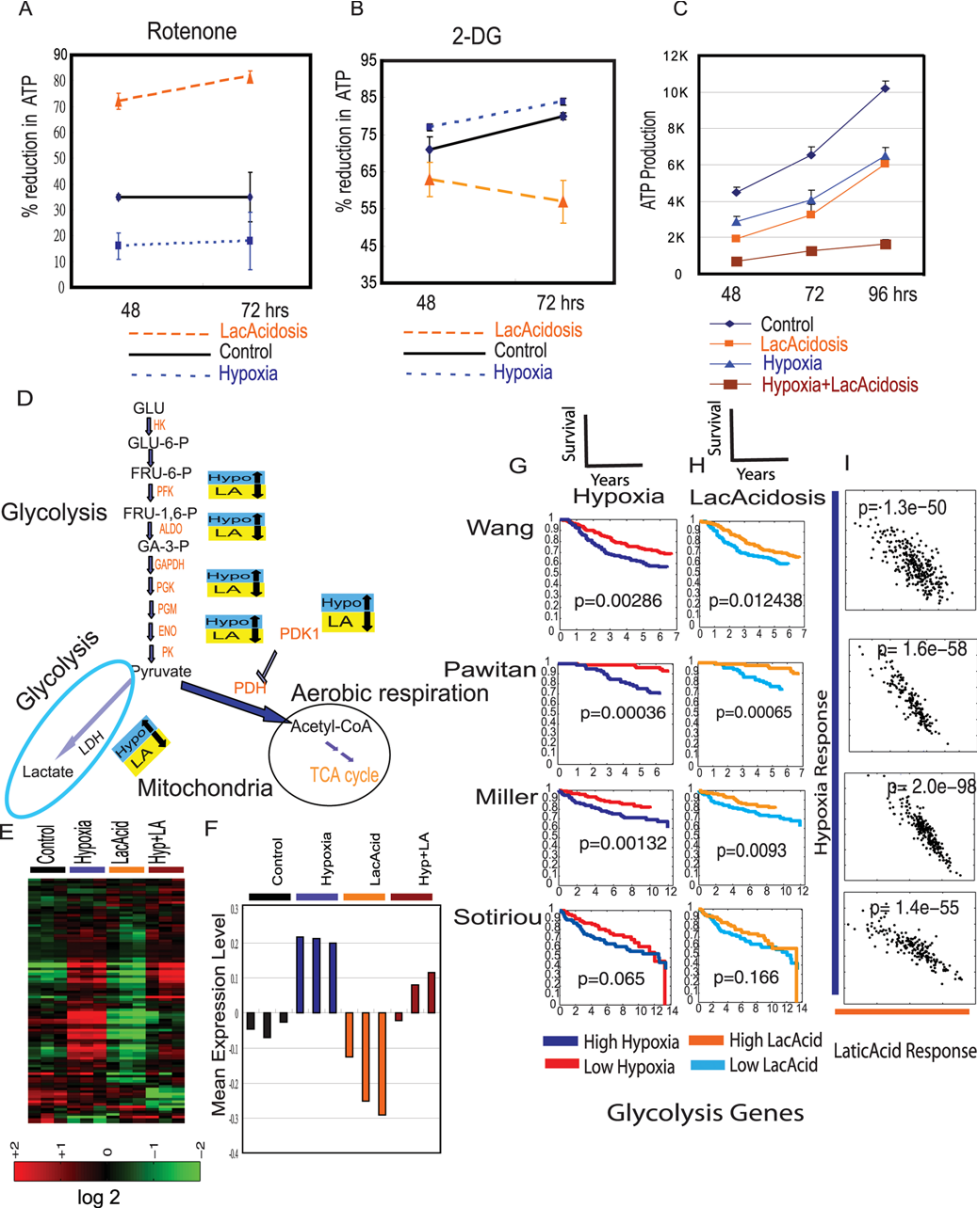
Lactic acidosis directs toward aerobic respiration by inhibiting the expression of glycolytic genes (A,B). The contribution of aerobic respiration and glycolysis to ATP generation under control, lactic acidosis and hypoxia is measured by the degree of inhibition of ATP generation after treatment of rotenone (A) and 2-DG (B) at the indicated time after treatment. (C) The amount of ATP generation at different time points under the indicated conditions. (D) The genes in the glycolysis pathways were up-regulated by hypoxia and down-regulated by lactic acidosis. (E) The expression of genes listed as “glycolysis pathway” was extracted and clustered. (F) The mean expression values of the 53 glycolysis genes for each HMEC under hypoxia, lactic acidosis and hypoxia/lactic acidosis are calculated and shown. (G)(H) The expression of genes in the glycolytic pathways under hypoxia and lactic acidosis were used to predict the pathways activity and stratified the indicated breast cancer samples. This small set of genes recapitulated the result using the whole lactic acidosis and hypoxia gene signatures. (I) Scatter plots showing the relationship between the probability of hypoxia response (Y-axis) and lactic acidosis response (X-axis) for genes in the glycolysis pathways. Each point in the scatter plots represents a single tumor from the indicated breast cancer data sets. The probability (p) between hypoxia and lactic acidosis signatures across all samples in the indicated data set is shown.

To investigate the possibility that lactic acidosis directly modulates the balance of energy production, we measured its influence on ATP production in cultured cells when aerobic respiration is inhibited by rotenone. In control conditions without hypoxia or lactic acidosis, we found that approximately 35 %of ATP production in Siha cells is sensitive to rotenone at 48 hours (Figure 5A). Under hypoxia, only 16% of ATP production was sensitive to rotenone at 48hours, reflecting the increased use of glycolysis for energy generation when oxygen is limited (Figure 5A). In contrast, this balance is dramatically changed under lactic acidosis with 72 % ATP production sensitive to rotenone. This effect is even more dramatic at 72 hours – while 35% of ATP production was sensitive to rotenone inhibition under control conditions, this increased to 82% under lactic acidosis and decreased to 18% under hypoxia (Figure 5A). We also tested the contribution of ATP from glycolysis with 2-DG. At 48hours, we found that 71 %of ATP production is sensitive to 2-DG. This is increased to 77% under hypoxia and reduced to 63% under lactic acidosis (Figure 5B). These results indicate that lactic acidosis redirects energy production toward the aerobic respiration *in vitro*, which may explain why lactic acidosis response can identify tumors with higher level of aerobic respiration.

These data reveal the distinct manner by which lactic acidosis and hypoxia redirect energy utilization – hypoxia favors the glycolytic pathways while lactic acidosis favors the aerobic respiration. This suggests these two stresses may impact cellular metabolism in distinct manner and have synergic effects on ATP inhibition when cells are exposed to simultaneous hypoxia and lactic acidosis. We measured ATP production under control, lactic acidosis, hypoxia and combined hypoxia and lactic acidosis. We found that lactic acidosis and hypoxia reduced the ATP production to ∼50% and 63% respectively in 48 and 96 hours (Figure 5C). This result indicates that while lactic acidosis also caused a reduction in energy production similar to hypoxia. When the cells were exposed to both hypoxia and lactic acidosis, ATP production was dramatically decreased to about 17.3% (on average) of the control cells (Figure 5C).

### Gene Expression Change Involving Energy Production in Hypoxia and Lactic Acidosis

To further understand how lactic acidosis and hypoxia modulate the balance between the aerobic respiration and glycolysis, we mapped their respective effects on gene expression onto the framework of metabolic pathways in energy metabolism (Figure 5D, E). From this analysis, we found that most genes in the glycolytic pathways, including PFK, ALDO, GADPH, PGK, PGM, ENO and PK, were significantly induced by hypoxia as expected from previous studies [21] (Figure 5D, E). Hypoxia induced the expression of LDHs, the enzymes required for the conversion of pyruvate to lactate. Hypoxia also induced the expression of gene encoding pyruvate dehydrogenase kinase 1 (PDK1), which inactivates the PDH by phosphorylation and prevents the conversion of pyruvate into acetyl-CoA, the essential substrate for TCA cycles during the aerobic respiration [41]. The PDK1 induction under hypoxia directs energy generation towards glycolysis instead of aerobic respiration [41]. Similarly, the average expression of these glycolysis genes was induced under hypoxia (Figure 5F). When these glycolysis genes are analyzed for their Gene Ontology enrichment, the top GO terms, as expected, are processes involved in the glycolysis and glucose metabolism (Table S8).

In contrast, expression levels of these glycolytic genes were consistently repressed by lactic acidosis (Figure 5D, E). For example, the expression levels of phosphofructokinase, fructose-1.6-bisphophatase and lactate dehydrogenase were all induced by hypoxia and repressed by lactic acidosis (Figure 5E). Lactic acidosis reduced the expression levels of all these genes from both the baseline level under normoxia and induced levels under hypoxia (Figure 5F). The changes in expression levels of these glycolysis genes are also shown (Table S9). Overall, hypoxia-induced change in gene expression favors the utilization of glycolytic pathways for energy generation whereas lactic acidosis represses the glycolysis process and favors the use of aerobic respiration as a mode of energy production, consistent with our experimental data of higher reliance on aerobic respiration (Figure 5A).

Under hypoxia, there was also a noticeable reduction in the expression of the genes in the TCA cycles and other mitochondria genes essential for aerobic respiration, consistent with previous studies [42],[43]. Lactic acidosis, by contrast, has no significant effect on their expression levels. The *in vivo* relevance of these changes in energy production by hypoxia and lactic acidosis was tested by examining survival predictions based on signatures of these two small sets of genes representing glycolysis and the TCA cycle. When the hypoxia-induced changes of glycolysis are projected into the same breast cancer expression studies, we found that high glycolysis pathway activities have prognostic significance similar to the significance of the whole hypoxia gene signatures (Figure 5G). On the other hand, the tumors with strong hypoxia-induced TCA pathways have entirely opposite effects – they have significantly better clinical outcomes (Figure S8A). Further, tumors with the high correlation between the glycolysis genes with lactic acidosis have better survival (Figure 5H), similar to the overall lactic acidosis gene signatures. Among the glycolysis genes, the lactic acidosis response correlated negatively with the hypoxia response in all four expression studies (Figure 5I). The lactic acidosis-induced changes in TCA cycle genes, on the other hand, have no predictive value for clinical outcomes (Figure S8B).

### Integrative Genomic Analysis Revealed that Lactic Acidosis Inhibits Akt Activities

**Figure 6:**
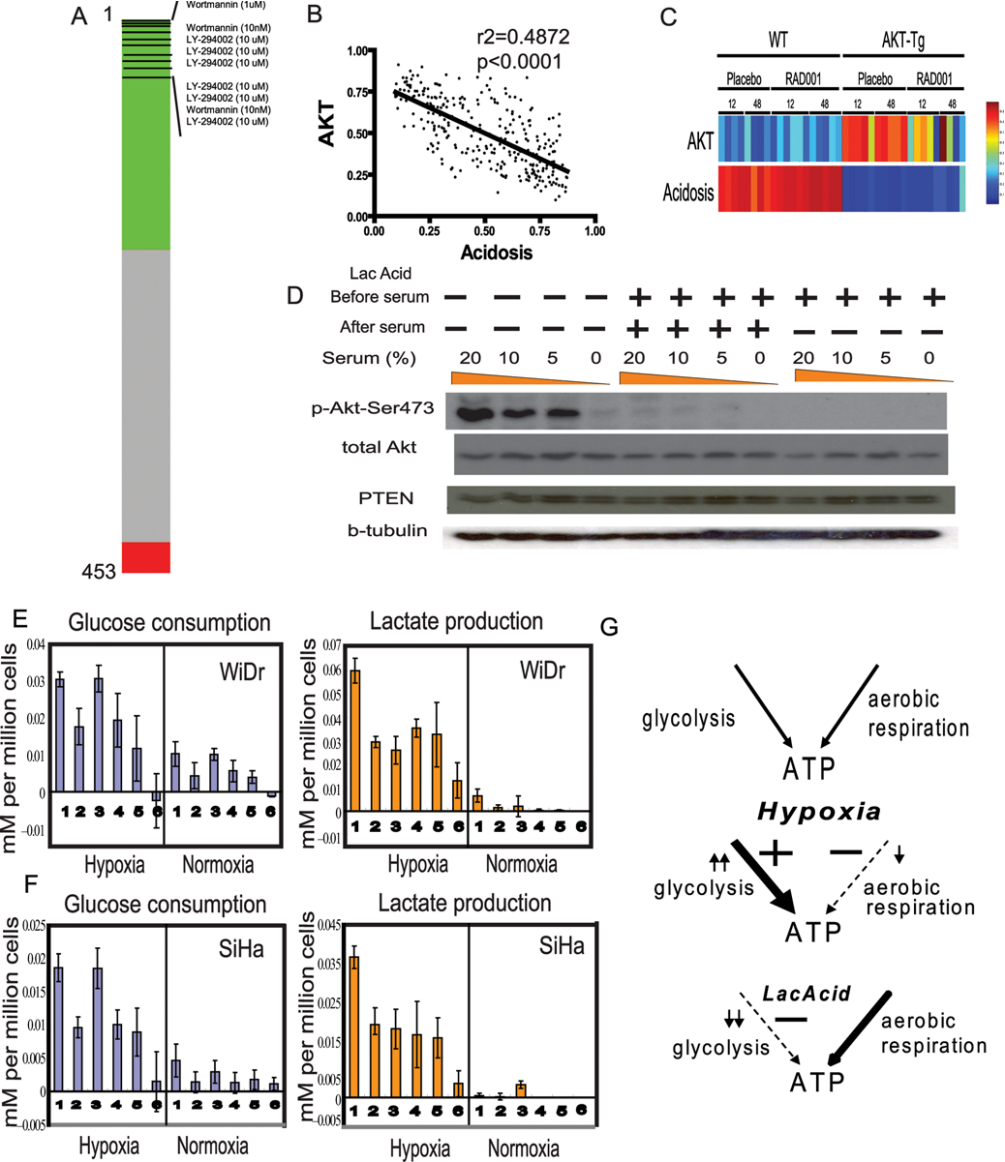
Lactic acidosis inhibits Akt and glycolytic phenotypes of cancer cells. (A) PI3K inhibitors are highly ranked with lactic acidosis signature in the connectivity map analysis. The “barview” is constructed from 453 horizontal lines, each representing an individual treatment instance, ordered by their corresponding connectivity scores calculated with lactic acidosis signature (+1, top; −1, bottom) with the instances corresponding to wormannin and LY-294002 were shown as black bars. Colors applied to the remaining instances reflect the sign of their scores (green, positive; gray, null; red, negative). (B), (C) The relationship between the predicted Akt and Acidosis pathway activities in the gene expression pattern of a breast cancer expression studies (B) and prostate tissue is shown between wild (WT) and Akt transgenic mouse (AKT-Tg) treated with placebo or mTOR inhibitor RAD001. (D) The effect of lactic acidosis on Akt activation in DU145 cells during serum exposure. Indicated amount of serum are added to the DU145 which have been placed in 0.2% serum conditions for 24 hours without (−) or with (+) 25 mM lactic acid. The same amount of cell lysates of DU145 cultured under indicated conditions were separated, transferred to blot and probed with indicated antibodies. (E), (F) The amount (mM) of lactate production (orange) and glucose consumption (blue) in 48 hour per million of WiDr (E) and SiHa (F) cells under hypoxia (left) or normoxia (right) with the following media conditions (1) control, (2) 25 mM lactic acidosis (pH 6.7), (3) 25 mM sodium lactate, (4) pH 6.7, (5) pH 6.5, (6) pH 6.0. (G) The model of the differentially modulated the balance of glycolysis and aerobic respiration as means of energy generation under control, hypoxia and lactic acidosis.

To further explore the signal transduction pathways of lactic acidosis through genomic analysis, we compared the lactic acidosis gene signatures with the database of “connectivity map” [44] composed of the gene expression patterns elicited by 453 different perturbations caused by 164 distinct small molecules. We assessed the similarity between lactic acidosis response and each reference expression profile in the data set with a nonparametric, rank-based pattern-matching strategy based on the Kolmogorov-Smirnov statistic [44]. From this analysis, the top perturbations positively correlated with the lactic acidosis gene signatures were different treatments of Wortmannin and LY-294002, two known inhibitors of phosphoinositide 3-kinases (PI3 kinase) (Figure 6A, detailed in Table S10). PI3 kinase phosphorylates phosphoinositides PtdIns(3,4)P2 (or “PIP2”) to PtdIns(3,4,5)P3 (or “PIP3”) molecule, which in turn recruit Akt to the cell membrane and trigger Akt phosphorylation and activation. Akt activation, in turn, initiates a cascade of cellular events from glucose uptake, energy utilization, cell growth and proliferation to survival and motility, that drive oncogenesis and tumour progression [45]. This association between acidosis and PI3K inhibition is consistent with the observations seen previously in muscle cells [46].

Gene signatures representing different pathways can be evaluated with lactic acidosis response signature in the gene expression data sets of breast cancer to further elucidate the molecular mechanisms of lactic acidosis [20],[21]. We evaluated this using several gene signatures reflecting oncogenic pathway deregulation induced by genetic manipulations [20]. In the three expression studies of breast cancers [35],[36],[47], we found a consistent inverse relationship (p<0.0001) between the acidosis and Akt pathway signatures – tumors with strong acidosis response tend to manifest low Akt pathway activities while tumors with low acidosis response manifest high Akt pathway activities (Figure 6B and Table S11). This inverse relationship was further noted in an independent mouse model of prostate neoplasia with overexpression of constitutively activated Akt [48] – the acidosis response is high in the normal prostate and low in the prostate of the Akt transgenic mouse which exhibit sign of prostate cancers (Figure 6C).

This inverse correlation between the lactic acidosis and Akt pathway activity in the tumor expression data lead us to hypothesize that lactic acidosis can inhibit the Akt pathway in the tumor cells. This possibility is also consistent with the correlation between lactic acidosis response and PI3K inhibition noted in the connectivity map analysis. Since this observed pathway activity seen in the gene expression may be caused by the corresponding change in the Akt enzymatic activity, we formally tested the effect of lactic acidosis on activation of Akt enzymatic activity in prostate cancer cell line DU145 during serum exposure. Growth of DU145 cells in a serum-free condition resulted in the inhibition of the Akt enzymatic activity, as seen by the absence of phosphorylation of Ser473 (Figure 6D). Upon exposure to serum, the Akt enzymatic activity became activated in 30 minutes, as indicated by the phosphorylation of Ser473 [49] (Figure 6D). However, this serum-induced Akt activation was abolished when the cells were simultaneously exposed to lactic acidosis during serum exposure (Figure 6D). Even after lactic acidosis exposure removed once media was changed to neutral pH during serum exposure, this Akt inhibition still persisted (Figure 6D). This suggests that Akt enzymatic and inferred pathway activity can be abolished by prior exposure to lactic acidosis.

### Lactic Acidosis Inhibits the Glycolytic Phenotypes of Cancer Cells

Given the important role of the PI3K/Akt pathway in the tumor glycolytic phenotypes [50], we explored whether lactic acidosis, with its ability to inhibit Akt pathways, can inhibit glycolysis phenotypes of cancer cells. This possibility is further supported by the repression of gene expression in the glycolytic pathways and increased reliance on aerobic respiration in lactic acidosis. We examined the glucose consumption and lactate production in two cancer cell lines – WiDr (colon cancer cell) and SiHa (cervical cancer cell). When grown in a control environment (condition 1) with ambient air, colon cancer cell WiDr exhibit features of aerobic glycolysis with modest glucose consumption and lactate production (Figure 6E). While 25 mM sodium lactate (condition 3) alone did not affect the glucose consumption significantly, all the remaining conditions (including lactic acidosis (condition 2), acidosis at pH 6.7, pH 6.5 and pH 6.0 (condition 4, 5, 6, created by HCl) significantly decreased glucose consumption. The acidosis-induced decrease in glucose consumption is also accompanied by a corresponding decrease in lactate production. This shows that lactic acidosis and acidosis lead to a significant reduction in glycolysis. Both glucose consumption and lactate production were increased under hypoxic conditions (0.5% O_2_). This hypoxia-induced increase was also dramatically reduced when cells were placed under lactic acidosis and acidosis conditions (conditions 2, 4, 5, 6) but not lactosis. Similar effects in decreasing glucose consumption and lactate production were also seen for SiHa (Figure 6F) and mouse MEF cells. This inhibition of glycolytic phenotypes by lactic acidosis can also explain the increased reliance on aerobic respiration as well as the ability of lactic acidosis response in identifying breast cancers with higher level of aerobic respiration.

Taken together, we propose a model (Figure 6G) in which lactic acidosis favors the utilization of aerobic respiration as the mode of energy production by inhibiting the glycolysis pathways. This is likely due to both the repression of expression of glycolysis genes and the inhibition of Akt enzymatic and pathway activities. This switch to aerobic respiration *in vitro* may also explain the ability of lactic acidosis gene signatures to identify breast cancers enriched in molecular pathways of aerobic respiration/mitochondria *in vivo* and more favorable clinical outcomes. Thus, the lactic acidosis gene signatures allow us to identify tumors with distinct metabolic profiles and clinical phenotypes. The reciprocal exchange of *in vitro* and *in vivo* global gene expression information via the common language of microarrays greatly enhanced our understanding of how individual microenvironmental stresses lead to relevant clinical phenotypes.

## Discussion

Lactic acidosis and hypoxia are two well recognized features in human cancers. Although tumor lactic acidosis is often thought to co-exist with hypoxia, relatively little is known about its cellular response, relationship with hypoxia or its role in tumor progression. Our current study presents, to our best knowledge, the first genomic analysis of lactic acidosis activities in human breast cancers *in vivo*. These analyses reveal that tumors exhibiting strong lactic acidosis and acidosis responses are associated with favorable clinical outcomes – in direct contrast to the poor clinical outcome associated with strong hypoxia response. Through various genomic analyses, this association with favorable outcomes is likely to be explained by the ability of lactic acidosis and acidosis to direct energy utilization toward aerobic respiration through the inhibition of glycolysis. Lactic acidosis mediates this effect both by inhibiting gene expression of glycolytic pathways and repressing Akt activation. In contrast to hypoxia, lactic acidosis represses the tumor “glycolytic” phenotype with the reduction of both glucose consumption and lactate production in tumor cells. Since tumor glycolysis is a crucial component of the malignant phenotypes and confers a significant proliferative advantage during somatic evolution [4], this “anti-Warburg” effect by lactic acidosis is likely to contribute to favorable clinical outcomes seen in tumors with strong lactic acidosis programs. The inhibition of Akt/glycolysis may also hamper the adaptive shift to anaerobic glycolysis under hypoxia and renders the cell vulnerable to energy depletion and cell death in hypoxic environments [13]. For example, lactic acidosis represses the LDH-A induction by hypoxia and LDH-A inhibition by RNAi has been previously shown to lead to poor tumor survival and diminished tumorgenicity [38].

A number of studies have now described the power in utilizing large scale gene expression data to develop signatures representing important biological states – in this context, the signature becomes a surrogate phenotype that can be used to explore the biological relevance in the diverse space of *in vitro* and *in vivo* systems, including human tumors [20],[51]. In this study, we have taken this approach further to bring signatures together to develop a mechanistic understanding of a clinically important biological process – lactic acidosis response. By investigating its relationship with various known molecular processes in the space of gene expression, we uncovered a positive association with a different mode of metabolic status (aerobic respiration and fatty acid/amino acid metabolism) and an inverse relationship with the PI3K/Akt pathways. These findings reveal the previously undefined complexity of lactic acidosis response program. These *in vitro* observations, in turn, help to explain the favorable clinical outcomes associated with strong lactic acidosis response *in vivo*. This use of gene expression data as common phenotypes to facilitate the reciprocal exchange of information between experimental perturbations *in vitro* and clinical phenotypes *in vivo* has greatly enhanced our understanding of the lactic acidosis response. We expect this approach is generally applicable and will broadly enhance our understanding in other biological processes. This unexpected result demonstrates the importance of analyzing individual microenvironmental factors separately to determine their respective contributions to tumor phenotypes.

One of the principal metabolic properties of cancer cells is their preferential use of glycolysis for energy generation even in the presence of oxygen (aerobic glycolysis). Warburg has speculated that this is caused by defective or functionally impaired mitochondria. Recent studies suggest that mutations affecting mitochondrial DNA or enzymes of the TCA cycle might contribute to tumor formation, tumor progression and the Warburg effect. Two enzymes in the TCA cycle enzymes, SDH and FH, are found to be tumor suppressor genes [52]. Given the importance of the choice of glycolysis vs. aerobic respiration for energy production in determining the tumor phenotypes and clinical outcomes of cancer patients, the regulatory circuits affecting for this choice have become subjects of intense investigation. For example, hypoxia triggers coordinated changes toward glycolysis through both the induced expression of genes encoding glucose transporters and glycolytic enzymes and repression of mitochondrial function [42],[43]. Hypoxia also induces the expression of PDK1. This phosphorylates and inactivates pyruvate dehdrogenase (PDH), which converts pyruvate to acetyl-CoA. In addition, Akt activation also favors glycolysis since it increases glucose transport and makes cancer cells dependent on glucose for their survival [50]. The cellular rate of glycolysis is also regulated on many levels, such as the availability of oxygen/substrate, NAD/NADH, AMP/ATP ratio, enzyme modification and allosteric inhibition. Other regulators, in contrast, can direct the cells toward aerobic respiration and suppress cancer phenotypes. For example, the tumor suppressor gene p53 can redirect toward aerobic respiration by both inducing genes required for aerobic respiration [39] and repressing genes inhibiting glycolysis [40]. Since the accumulation of lactic acidosis comes from the excess of product from the glycolysis, the inhibition of glycolysis by lactic acidosis can be thought of as a negative feedback. Extracellular acidity is determined both by the abundance of different acidic substances (e.g. lactic acid, CO_2_) [53] and the buffering capacity of the extracellular fluid. This negative feedback mechanism may allow cells to avoid acidity-induced cell death by adjusting the rate of glycolysis based on extracellular pH. Given the association of lactic acidosis pathway activity with favorable clinical outcomes, understanding the mechanism by which lactic acidosis inhibits Akt and glycolysis may lead to novel therapeutic strategies to modify tumor behavior. The lactic acidosis gene signature can also be used to identify cancers preferentially using aerobic respiration and therefore likely to respond better to chemotherapeutics targeting mitochondrial functions [54].

In addition to generation of ATP, glycolysis is responsible for the generation of acetyl-CoA, which feeds into the TCA cycle for aerobic respiration. Thus, the reduction in glycolysis under lactic acidosis may imply a reduction in the amount of acetyl-CoA derived from the glycolytic process. To maintain cellular energy, cells may increase the use of other energy sources, such as the β-oxidation of fatty acids as source of acetyl-CoA. Since Akt pathway activity is known to suppress β-oxidation and thus energy generation from fatty acids [50],[55], Akt inhibition by lactic acidosis may increase β-oxidation of fatty acid and thereby compensate for the increased demand of acetyl-CoA for energy generation. In support of this change in metabolic state, breast cancers with strong lactic acidosis responses are highly enriched in gene sets of fatty acid degradation and amino acid metabolism (GSEA analyses). There is increased expression of PPAR-alpha and several other genes mediating fatty acid oxidation in the lactic acidosis response. Together, these *in vivo* and *in vitro* observations indicate that lactic acidosis induces extensive and coordinated changes in the mode of metabolism and energy utilization, which in turn may account for their effects on the clinical phenotypes of breast cancer.

These data suggest that the lactic acidosis-induced gene expression program may have a direct causal role in impacting tumor biology to affect clinical outcomes. It will be important to understand what specific lactic acidosis-driven biological processes underlie the phenotypic differences between groups of tumors separated by lactic acidosis pathway activity. The mechanisms underlying the variation in lactic acidosis responses in breast are still unknown. There are three reasonable possibilities; variations in the lactic acidosis-response program could reflect: 1) actual variations in lactate and/or acidity in the tumors 2) cell-type-specific variations in the magnitude of, or threshold for, the response to *bona fide* lactate and/or acidity in tumors or 3) inappropriate activation of the lactic acidosis response resulting from genetic and/or epigenetic alterations in cancers. Given these possibilities, it is important to note that several previous studies have suggested that high level of tumor lactate is associated with poor clinical outcomes in several solid tumors [56],[57],[58]. This is in contrast to our results based on the analysis of lactic acidosis gene signatures, but is not necessarily contradictory as the two may be decoupled in tumors. It is relevant to note that acidosis was able to impose strong selection pressure and likely contributes to the observed responses seen in a group of tumors [4],[14],[18]. This difference highlights the distinction between the measured physiological parameters (pO2, lactate and acidity) and observed cellular responses (hypoxia and lactic acidosis response genes) in human cancers. For example, the expression of carbonic anhydrase IX (CA9) expression is induced by hypoxia. Therefore, CA9 is frequently used as an “endogenous” marker for low tumor pO2 – *bona fide* hypoxia [59]. But tumor pO2 and hypoxia response are two distinct measurements which may not exhibit tight correlation in all instances. Although many studies have concluded a low tumor pO2 is associated with CA9 expression, other studies point out the lack of direct correlation between CA9 expression and pO2 [60],[61],[62]. In other studies, high levels of tumor lactate [56],[63] and acidic pH [64] are usually associated with tumor metastasis or poor treatment response in solid tumors. The discrepancy between high lactate in tumors as a predictor of poor prognosis and strong lactic acidosis response as a predictor of good prognosis strongly suggests a likely disentanglement between measured physiological parameters and observed cellular responses. This discrepancy may be due to continuous fluctuations in the physiological parameters, different turnovers in the mRNA/protein, cell type-specific variations in the responses or it may result from genetic and/or epigenetic alterations in cancers unrelated to the physiological parameters. It is therefore important to determine whether these variations in the lactic acidosis response program are associated with actual variations in lactate and acidity in the tumors or result from other causes without significant connections to tumor lactate or acidity [65].

It is widely believed that the intimate relationship between tumor progression, tumor microenvironment and the response of the tumor to that environment, when combined with other genetic and clinical factors, offers promise for improving our understanding of the heterogeneity of the disease. Progress in this direction will require a substantial advance in our – currently limited – ability to dissect the roles played by multiple characteristics of the tumor microenvironment. We aim to develop this approach to further dissect other various microenvironmental factors in cancers, such as glucose starvation, reoxygenation and ATP depletion [1]. The availability of this information will allow researchers to gradually establish an integrative metabolic profile of human cancers. Additionally, diverse genetic and molecular characteristics from clinical data will help further elucidate heterogeneous properties of tumors and lead to targeting treatments with better prognosis [26].

## Materials and Methods

### Cell Culture and Conditions Reflecting Different Environmental Stresses

Human mammalian epithelial cells (HMEC) were cultured in MEGM (Cambrex) and growth factors were withdrawn for 24 hrs before being placed under different environmental stresses. DU145 cells were cultured in RPMI1640 with 10% FBS, 1% sodium pyruvate, 1% L-glutamine, 1% Hepes and 1% antibiotics (penicillin, 10000 UI/ml; streptomycin, 10000 UI/ml). WiDr and SiHa cells were cultured in DMEM with 10%FBS. Lactic acidosis conditions were created with the addition of 25 mM lactic acid (Sigma) to pH 6.7. Hypoxia was created by lowering the oxygen level to 2%. Similarly, the lactosis condition was created with the addition of 25 mM sodium lactate, while acidosis conditions were created by titrating media to pH of 6.7 with HCl.

### RNA Isolation and Microarray Analysis

RNAs were extracted by miRVana kits (Ambion) and hybridized to Affymetrix Hu133 plus 2 genechips with standard protocol. All microarray data are available on GEO (GEO accession number GSE9649). Hierarchical clustering with weighted average linkage clustering was performed after indicated data filtering based on spot quality and variations in signal intensity as described [66]. The analyses of microarrays from the lactic acidosis/hypoxia and acidosis/lactosis experiments were performed using a sparse ANOVA modeling framework outlined and implemented in the software package, Bayesian Factor Regression Models (BFRM)[67] with detailed parameter files and normalized RMA data available in the supplementary section. In the context of a designed experiment (having controls and experimental groups) BFRM provides a probability of differential expression for each gene and each experimental group. A signature corresponding to each experimental group was compiled by listing those genes with high probability of differential expression as well as high levels of fold change in expression. Principal components were then used to compute weights for each gene such that the weighted average of expression levels showed a clear ability to distinguish the relevant group from others in the experiment. Expression levels of tumor samples on each of the signatures were calculated according to the weighted average obtained from this principal components analysis and after subtraction of mean expression and doping control correction factors. In order to distinguish differential survival associated with expression level of a particular signature, the patient population was split into high and low expression level groups and Kaplan-Meyer survival curves were computed for each group.

### Real Time RT-PCR

RNAs were reverse-transcribed to cDNAs with SuperScript II reverse transcription kit following the manufacturer's protocol (Invitrogen). cDNAs were then used as the substrate for gene expression level measurements by qPCR with Power SYBRGreen PCR Mix (Applied Biosystems) and primers specific for ErbB3(Forward:CAGGGGTGTAAAGGACCAGA, Reverse:CGCCAGTAGAGAAAAGTGCC), CD55(Forward:AGGTCCCACCAACAGTTCAG, Reverse:AAAATGCTTGGTTGTCCTGG), PLAU(Forward:TGTGAGATCACTGGCTTTGG, Reverse:ACACAGCATTTTGGTGGTGA), SOD2(Forward:TTTGGGGACTTGTAGGGATG, Reverse:AGAAAGCCGAGTGTTTCCCT), Actin-beta(Forward:CTCTTCCAGCCTTCCTTCCT, Reverse:AGCACTGTGTTGGCGTACAG), B2M(Forward:TGCTGTCTCCATGTTTGATGTATCT, Reverse:TCTCTGCTCCCCACCTCTAAGT) respectively following the manufacturer's protocol (Applied Biosystem).

### Serum Stimulation of Akt Activation and Western Blot Analysis

DU145 cells were serum-starved (0.2%FBS) for 24 hrs, followed by 24 hr of continuous incubation in serum starved (0.2%FBS) media (as the control) and media with 25 mM lactic acid. 20%, 10%, and 5% FBS were applied for 30 mins to induce Akt activation. Proteins were extracted with PARIS kit (Ambion) and equal amount of protein samples were loaded to SDS-PAGE gels and blotted with pSer473 Akt antibody (Cell signaling) and other indicated antibodies.

### Glucose and Lactate Measurement

WiDr and SiHa cells were plated in six-well dishes (800,000 cells per well). The next day fresh media of respective conditions, including control, 25 mM lactic acidosis, 25 mM sodium lactate, acidosis of pH 6.7, pH 6.5 and pH 6.0 were applied to cells with the continuous incubation of 48 hrs under either normoxia or hypoxia (0.5% O_2_). After 48hr incubation, media were collected for glucose (ACCU-CHECK, Roche) and lactate (ARKRAY) measurements and normalized against cell number to obtain the glucose consumption/lactate production per million cells.

### ATP Determination

SiHa cells were plated at the density of 2×10^4^ cells/ml. On the next day, respective media of control and 25 mM lactic acidosis, as well as media containing drug inhibitors, 2-DG and rotenone (Sigma), would be applied. They will then be incubated under normoxia and hypoxia (1% oxygen) respectively. ATP was measured by ATPlite 1 step luminescence ATP detection assay system kit with the protocol provided by the manufacturer after 48 and 72 hours (Perkin Elmer). To prevent the interference caused by different colors of control versus lactic acidosis media, we replaced culture media with PBS right before the addition of substrate solution.

## Supporting Information

Figure S1 The pathway activities of hypoxia (A) and lactic acidosis (B) signatures were estimated as probability (Y-axis) by binary regression models in the HMECs exposed to indicated treatments (control, lactic acidosis, hypoxia and combined lactic acidosis and hypoxia, X-axis).
(1.02 MB EPS)

Figure S2 The change in gene expression in the indicated conditions for a set of genes whose expression is induced (A) or repressed (B) only in the presence of both hypoxia and lactic acidosis.
(3.02 MB EPS)

Figure S3 The confirmation of the induction of ERBB3 and SOD2 expression by acidosis using real time RT-PCR.
(1.09 MB EPS)

Figure S4 The prognostic values of gene signatures reflecting hypoxia (A, C) and lactic acidosis (B, D) response among the patients treated (A, B) and untreated (C, D) with tamoxifen in the Sotiriou datasets. The graphs show Kaplan-Meier curves for two patient subsets simply split on the median signature level in each case, for visual presentation. The p-values are for regression coefficients of the signature in the survival model analysis.
(0.93 MB EPS)

Figure S5 Prognostic values of predictive model based on different combination of gene signatures and clinical variables. The breast cancers are separated in (A) based on hypoxia, lactic acidosis response and nodal status; in (B) based on hypoxia, lactic acidosis response, tumor size, ER status and nodal status; in (C) tumor size, ER status and nodal status. The graphs show Kaplan-Meier curves for two patient subsets simply split on the median signature level in each case, for visual presentation. The p-values are for regression coefficients of the signature in the survival model analysis.
(1.31 MB EPS)

Figure S6 Genes in the TCA cycle gene set are highly enriched in the tumors with high lactic acidosis using GSEA.
(0.02 MB PNG)

Figure S7 Breast samples in the Pawitan data set arranged from left to right by descending hypoxia score (top row). The expression of genes in TCA cycles in these samples is shown in heat map, orange color being higher expression while blue color being lower expression, together with hypoxia score.
(0.57 MB EPS)

Figure S8 Higher hypoxia response of TCA cycle genes was associated with better prognosis, in contrast to the association with poor prognosis for hypoxia genelists (A). On the other hand, TCA cycle genes of lactic acidosis response were not associated with differences in clinical outcome (B).
(1.87 MB EPS)

Table S1 Estimated posterior probability and corresponding Bayesian p-value (1-probability) for gene probe sets showing responses to hypoxia, lactic acidosis and acidosis.
(10.86 MB XLS)

Table S2 GO terms enrichments (with p values) for genes which are upregulated and downregulated in HMECs under lactic acidosis.
(0.06 MB XLS)

Table S3 GO terms enrichments (with p values) for genes which are upregulated and downregulated in HMECs under hypoxia.
(0.03 MB XLS)

Table S4 The comparison between GO term enrichment under hypoxia and lactic acidosis.
(0.02 MB XLS)

Table S5 The expression mean-centered values of indicated MCT family proteins under hypoxia and lactic acidosis.
(0.02 MB XLS)

Table S6 The lists of genes which are strongly induced or repressed (top 1% probability) under lactic acidosis and hypoxia.
(0.18 MB XLS)

Table S7 Gene sets enriched in the four expression datasets of breast cancers with high vs. low lactic acidosis pathways from the GSEA analysis.
(0.05 MB XLS)

Table S8 The GO terms enriched for genes used for the analysis of glycolysis pathways.
(0.02 MB TXT)

Table S9 p-values for the changes in gene expression under lactic acidosis and hypoxia for the listed glycolysis genes.
(0.02 MB XLS)

Table S10 Connectivity map analysis of the lactic acidosis gene signatures.
(0.09 MB XLS)

Table S11 Relationship between lactic acidosis signature and Akt pathway activities in different tumor expression datasets.
(0.04 MB XLS)

Text S1 Statistical supplement. Details of Bayesian factor regression models and survival analyses.
(0.14 MB PDF)

## References

[1] Vaupel P (2004). Tumor microenvironmental physiology and its implications for radiation oncology.. Semin Radiat Oncol.

[2] Helmlinger G, Yuan F, Dellian M, Jain RK (1997). Interstitial pH and pO2 gradients in solid tumors in vivo: high-resolution measurements reveal a lack of correlation.. Nat Med.

[3] Vaupel P, Hockel M (2000). Blood supply, oxygenation status and metabolic micromilieu of breast cancers: characterization and therapeutic relevance.. Int J Oncol.

[4] Gatenby RA, Gillies RJ (2004). Why do cancers have high aerobic glycolysis?. Nat Rev Cancer.

[5] Cardone RA, Casavola V, Reshkin SJ (2005). The role of disturbed pH dynamics and the Na+/H+ exchanger in metastasis.. Nat Rev Cancer.

[6] Semenza GL (2002). HIF-1 and tumor progression: pathophysiology and therapeutics.. Trends Mol Med.

[7] Harris AL (2002). Hypoxia–a key regulatory factor in tumour growth.. Nat Rev Cancer.

[8] Huang WC, Swietach P, Vaughan-Jones RD, Ansorge O, Glitsch MD (2008). Extracellular acidification elicits spatially and temporally distinct Ca2+ signals.. Curr Biol.

[9] Xu L, Fidler IJ (2000). Acidic pH-induced elevation in interleukin 8 expression by human ovarian carcinoma cells.. Cancer Res.

[10] Fukumura D, Xu L, Chen Y, Gohongi T, Seed B (2001). Hypoxia and acidosis independently up-regulate vascular endothelial growth factor transcription in brain tumors in vivo.. Cancer Res.

[11] Shi Q, Le X, Wang B, Abbruzzese JL, Xiong Q (2001). Regulation of vascular endothelial growth factor expression by acidosis in human cancer cells.. Oncogene.

[12] Mekhail K, Gunaratnam L, Bonicalzi ME, Lee S (2004). HIF activation by pH-dependent nucleolar sequestration of VHL.. Nat Cell Biol.

[13] Graham RM, Frazier DP, Thompson JW, Haliko S, Li H (2004). A unique pathway of cardiac myocyte death caused by hypoxia-acidosis.. J Exp Biol.

[14] Moellering RE, Black KC, Krishnamurty C, Baggett BK, Stafford P (2008). Acid treatment of melanoma cells selects for invasive phenotypes.. Clin Exp Metastasis.

[15] Zieker D, Schafer R, Glatzle J, Nieselt K, Coerper S (2008). Lactate modulates gene expression in human mesenchymal stem cells.. Langenbecks Arch Surg.

[16] Nowik M, Lecca MR, Velic A, Rehrauer H, Brandli AW (2008). Genome-wide gene expression profiling reveals renal genes regulated during metabolic acidosis.. Physiol Genomics.

[17] Fang JS, Gillies RD, Gatenby RA (2008). Adaptation to hypoxia and acidosis in carcinogenesis and tumor progression.. Semin Cancer Biol.

[18] Gatenby RA, Gillies RJ (2008). A microenvironmental model of carcinogenesis.. Nat Rev Cancer.

[19] Laconi E (2007). The evolving concept of tumor microenvironments.. Bioessays.

[20] Bild AH, Yao G, Chang JT, Wang Q, Potti A (2005). Oncogenic pathway signatures in human cancers as a guide to targeted therapies.. Nature.

[21] Chi JT, Wang Z, Nuyten DS, Rodriguez EH, Schaner ME (2006). Gene Expression Programs in Response to Hypoxia: Cell Type Specificity and Prognostic Significance in Human Cancers.. PLoS Med.

[22] Lamb J, Ramaswamy S, Ford HL, Contreras B, Martinez RV (2003). A mechanism of cyclin D1 action encoded in the patterns of gene expression in human cancer.. Cell.

[23] Chang HY, Sneddon JB, Alizadeh AA, Sood R, West RB (2004). Gene Expression Signature of Fibroblast Serum Response Predicts Human Cancer Progression: Similarities between Tumors and Wounds.. PLoS Biol.

[24] Sorensen BS, Hao J, Overgaard J, Vorum H, Honore B (2005). Influence of oxygen concentration and pH on expression of hypoxia induced genes.. Radiother Oncol.

[25] Sorensen BS, Alsner J, Overgaard J, Horsman MR (2007). Hypoxia induced expression of endogenous markers in vitro is highly influenced by pH.. Radiother Oncol.

[26] Lucas J, Carvalho C, Wang Q, Bild A, Nevins J, Do Kea (2006). Sparse statistical modelling in gene expression genomics;.

[27] Seo DM, Goldschmidt-Clermont PJ, West M (2007). Of mice and men: Sparse statistical modelling in cardiovascular genomics.. Annals of Applied Statistics.

[28] Chang JT, Nevins JR (2006). GATHER: a systems approach to interpreting genomic signatures.. Bioinformatics.

[29] Adams DJ, Wahl ML, Flowers JL, Sen B, Colvin M (2006). Camptothecin analogs with enhanced activity against human breast cancer cells. II. Impact of the tumor pH gradient.. Cancer Chemother Pharmacol.

[30] Wahl ML, Pooler PM, Briand P, Leeper DB, Owen CS (2000). Intracellular pH regulation in a nonmalignant and a derived malignant human breast cell line.. J Cell Physiol.

[31] Mense SM, Sengupta A, Zhou M, Lan C, Bentsman G (2006). Gene expression profiling reveals the profound upregulation of hypoxia-responsive genes in primary human astrocytes.. Physiol Genomics.

[32] Chi J-T, Rodriguez EH, Wang Z, Nuyten DSA, Mukherjee S (2007). Gene Expression Programs of Human Smooth Muscle Cells: Tissue-Specific Differentiation and Prognostic Significance in Breast Cancers.. PLoS Genetics.

[33] Huang E, Ishida S, Pittman J, Dressman H, Bild A (2003). Gene expression phenotypic models that predict the activity of oncogenic pathways.. Nat Genet.

[34] Sotiriou C, Wirapati P, Loi S, Harris A, Fox S (2006). Gene expression profiling in breast cancer: understanding the molecular basis of histologic grade to improve prognosis.. J Natl Cancer Inst.

[35] Pawitan Y, Bjohle J, Amler L, Borg AL, Egyhazi S (2005). Gene expression profiling spares early breast cancer patients from adjuvant therapy: derived and validated in two population-based cohorts.. Breast Cancer Res.

[36] Minn AJ, Gupta GP, Siegel PM, Bos PD, Shu W (2005). Genes that mediate breast cancer metastasis to lung.. Nature.

[37] Subramanian A, Tamayo P, Mootha VK, Mukherjee S, Ebert BL (2005). Gene set enrichment analysis: a knowledge-based approach for interpreting genome-wide expression profiles.. Proc Natl Acad Sci U S A.

[38] Fantin VR, St-Pierre J, Leder P (2006). Attenuation of LDH-A expression uncovers a link between glycolysis, mitochondrial physiology, and tumor maintenance.. Cancer Cell.

[39] Matoba S, Kang JG, Patino WD, Wragg A, Boehm M (2006). p53 regulates mitochondrial respiration.. Science.

[40] Bensaad K, Tsuruta A, Selak MA, Vidal MN, Nakano K (2006). TIGAR, a p53-inducible regulator of glycolysis and apoptosis.. Cell.

[41] Kim JW, Tchernyshyov I, Semenza GL, Dang CV (2006). HIF-1-mediated expression of pyruvate dehydrogenase kinase: a metabolic switch required for cellular adaptation to hypoxia.. Cell Metab.

[42] Papandreou I, Cairns RA, Fontana L, Lim AL, Denko NC (2006). HIF-1 mediates adaptation to hypoxia by actively downregulating mitochondrial oxygen consumption.. Cell Metab.

[43] Zhang H, Gao P, Fukuda R, Kumar G, Krishnamachary B (2007). HIF-1 inhibits mitochondrial biogenesis and cellular respiration in VHL-deficient renal cell carcinoma by repression of C-MYC activity.. Cancer Cell.

[44] Lamb J, Crawford ED, Peck D, Modell JW, Blat IC (2006). The Connectivity Map: using gene-expression signatures to connect small molecules, genes, and disease.. Science.

[45] Vivanco I, Sawyers CL (2002). The phosphatidylinositol 3-Kinase AKT pathway in human cancer.. Nat Rev Cancer.

[46] Franch HA, Raissi S, Wang X, Zheng B, Bailey JL (2004). Acidosis impairs insulin receptor substrate-1-associated phosphoinositide 3-kinase signaling in muscle cells: consequences on proteolysis.. Am J Physiol Renal Physiol.

[47] Miller LD, Smeds J, George J, Vega VB, Vergara L (2005). An expression signature for p53 status in human breast cancer predicts mutation status, transcriptional effects, and patient survival.. Proc Natl Acad Sci U S A.

[48] Majumder PK, Febbo PG, Bikoff R, Berger R, Xue Q (2004). mTOR inhibition reverses Akt-dependent prostate intraepithelial neoplasia through regulation of apoptotic and HIF-1-dependent pathways.. Nat Med.

[49] Persad S, Attwell S, Gray V, Mawji N, Deng JT (2001). Regulation of protein kinase B/Akt-serine 473 phosphorylation by integrin-linked kinase: critical roles for kinase activity and amino acids arginine 211 and serine 343.. J Biol Chem.

[50] Elstrom RL, Bauer DE, Buzzai M, Karnauskas R, Harris MH (2004). Akt stimulates aerobic glycolysis in cancer cells.. Cancer Res.

[51] Nevins JR, Potti A (2007). Mining gene expression profiles: expression signatures as cancer phenotypes.. Nat Rev Genet.

[52] Gottlieb E, Tomlinson IP (2005). Mitochondrial tumour suppressors: a genetic and biochemical update.. Nat Rev Cancer.

[53] Helmlinger G, Sckell A, Dellian M, Forbes NS, Jain RK (2002). Acid production in glycolysis-impaired tumors provides new insights into tumor metabolism.. Clin Cancer Res.

[54] Costantini P, Jacotot E, Decaudin D, Kroemer G (2000). Mitochondrion as a Novel Target of Anticancer Chemotherapy.. J Natl Cancer Inst.

[55] Buzzai M, Bauer DE, Jones RG, Deberardinis RJ, Hatzivassiliou G (2005). The glucose dependence of Akt-transformed cells can be reversed by pharmacologic activation of fatty acid beta-oxidation.. Oncogene.

[56] Brizel DM, Schroeder T, Scher RL, Walenta S, Clough RW (2001). Elevated tumor lactate concentrations predict for an increased risk of metastases in head-and-neck cancer.. Int J Radiat Oncol Biol Phys.

[57] Walenta S, Wetterling M, Lehrke M, Schwickert G, Sundfor K (2000). High lactate levels predict likelihood of metastases, tumor recurrence, and restricted patient survival in human cervical cancers.. Cancer Res.

[58] Walenta S, Mueller-Klieser WF (2004). Lactate: mirror and motor of tumor malignancy.. Semin Radiat Oncol.

[59] Lal A, Peters H, St Croix B, Haroon ZA, Dewhirst MW (2001). Transcriptional response to hypoxia in human tumors.. J Natl Cancer Inst.

[60] Mayer A, Hockel M, Vaupel P (2005). Carbonic anhydrase IX expression and tumor oxygenation status do not correlate at the microregional level in locally advanced cancers of the uterine cervix.. Clin Cancer Res.

[61] Loncaster JA, Harris AL, Davidson SE, Logue JP, Hunter RD (2001). Carbonic anhydrase (CA IX) expression, a potential new intrinsic marker of hypoxia: correlations with tumor oxygen measurements and prognosis in locally advanced carcinoma of the cervix.. Cancer Res.

[62] Hedley D, Pintilie M, Woo J, Morrison A, Birle D (2003). Carbonic anhydrase IX expression, hypoxia, and prognosis in patients with uterine cervical carcinomas.. Clin Cancer Res.

[63] Schwickert G, Walenta S, Sundfor K, Rofstad EK, Mueller-Klieser W (1995). Correlation of high lactate levels in human cervical cancer with incidence of metastasis.. Cancer Res.

[64] Gerweck LE (1998). Tumor pH: implications for treatment and novel drug design.. Semin Radiat Oncol.

[65] Shin HJ, Kim JY, Yoo CW, Roberts SA, Lee S (2007). Carbonic anhydrase 9 (CA9) expression in tumor cells enhances sensitivity to tirapazamine.. J Cancer Res Clin Oncol.

[66] Eisen MB, Spellman PT, Brown PO, Botstein D (1998). Cluster analysis and display of genome-wide expression patterns.. Proc Natl Acad Sci U S A.

[67] Carvalho C, Chang J, Lucas J, Wang Q, Nevins JR (2007). High-dimensional sparse factor modeling: Applications in gene expression genomics.. Journal of American Statistical Society.

