# Supplementary figures and images for: The Genomic Analysis of Lactic Acidosis and Acidosis Response in Human Cancers
Source: PLoS Genet. 2008 Dec 5;4(12):e1000293. doi: 10.1371/journal.pgen.1000293 (PMC2585811; doi:10.1371/journal.pgen.1000293)

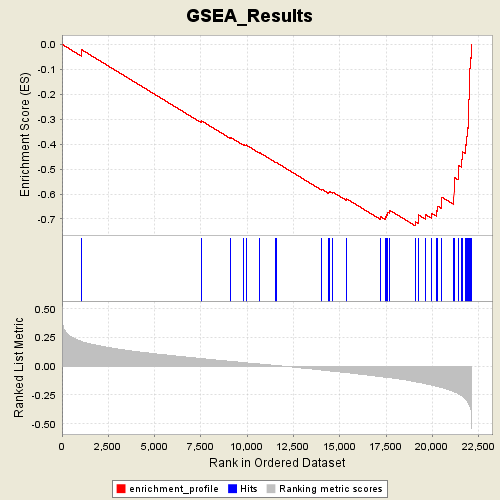

Supplement: Figure S6 — Genes in the TCA cycle gene set are highly enriched in the tumors with high lactic acidosis using GSEA. (0.02 MB PNG) [file pgen.1000293.s006.png]
