# Supplementary material for: The Genomic Analysis of Lactic Acidosis and Acidosis Response in Human Cancers
Source: PLoS Genet. 2008 Dec 5;4(12):e1000293. doi: 10.1371/journal.pgen.1000293 (PMC2585811; doi:10.1371/journal.pgen.1000293)
Supplement: Text S1 — Statistical supplement. Details of Bayesian factor regression models and survival analyses. (0.14 MB PDF) [file pgen.1000293.s020.pdf]

The genomic analysis of response to lactic acidosis in human cancers
# - Statistical supplement -

Joseph E Lucas[†] & Mike West[*,†]

joseph.lucas@duke.edu, mw@stat.duke.edu

[†]Institute for Genome Sciences & Policy
Duke University, Durham NC 27710

[*]Department of Statistical Science
Duke University, Durham NC 27708

May 22, 2008

Statistical model context

The analysis of the two sets of experimental intervention data to identify genes responsive to the several environmental stresses were performed using the Bayesian Factor Regression Models (BFRM) framework and associated software [1] that has been developed and utilised in a number of experimental and observational gene expression studies [2, 3]. In the simple designed experiments to explore the effects of hypoxia, lactic acidosis and so forth, BFRM provides a sparse Anova model framework, and also allows for model-based correction of all gene expression measures using the Affymetrix control/housekeeping probe sets on each array, as in [2]. This latter facility addresses experimental/assay artifactual effects on the summary RMA expression measures on a gene-sample specific basis. We used the same analysis – but now of course just involving the assay artifact terms – for each of the five breast cancer samples; in these five analyses, we simply aimed to correct the expression data for assay artifact prior to evaluating the *in vitro* defined expression signatures. The parameter input files for running BFRM in the analyses reported are available at the web site along with other supplementary materials.

Representing gene expression values as $x_{g,i}$, (gene g and sample i), the general model framework has

$$x_{g,i} \sim N(\sum_j \beta_{g,j} H_{i,j}, \psi_g),$$

where ψ_g is the gene $g-$specific residual/noise variance, $H_{i,j}$ $(j = 1 : k)$ represent known covariate values on sample i, and $\beta_g = (\beta_{g,1}, \ldots, \beta_{g,k})'$ is a column vector of regression coefficients on these covariates that is specific to gene g. The analysis uses the sparsity priors detailed in [1, 2] and implemented in BFRM. In analysis of the microenvironment stress response data, the covariates include dummy (0/1) variables (0=control cells, 1=stressed cells) and a set of five housekeeping control factors constructed as the first five principal components of the full set of control probesets on the arrays. For the five sets of breast cancer samples, we used the same model and covariates but, of course, there we have no stress parameters and the model is fitted simply to identify any

significant assay artifacts that are predicted by the control factors. We then work with the corrected data on the tumor samples:

$$y_{g,i} = x_{g,i} - \sum_j \pi^*_{g,j} \beta^*_{g,j} H_{j,i},$$

where $\pi^*_{g,j}$ is the posterior probability of a non-zero value of $\beta_{g,j}$ and $\beta^*_{g,j}$ is the estimated value of that parameter.

This statistical analysis computes, for all (47,000+) probe sets on the array, approximate summaries of the posterior distributions for each gene that include (but are not limited too): (a) an estimated mean expression level in the control cells, coupled with an estimate of the variance ψ_g of the purely random noise in expression about that level; (b) the estimated probability $\pi^*_{g,j} = Pr(\beta_{g,j}|\text{Data}) \neq 0$ for all g, j; (c) the estimate $\beta^*_{g,j} = E(\beta_{g,j}|\beta_{g,j} \neq 0, \text{Data})$ for each g, j.

Analysis of experimental data

For each of our two experiments on Human Mammary Epithelial Cells (HMEC), we used BFRM to compute the probability of a change in expression level for each gene and within each stress response group – simply the $\pi^*_{g,\cdot}$. The BFRM output provides these probabilities of a non-zero stress response for each gene, and the estimated changes in expression as a result of that response. We can select and rank genes according to their probabilities of stress responses; in the reported analyses we defined signature gene sets by choosing genes showing a stress response probability in excess of 0.99. Such genes are referred to as members of the corresponding pathway. This leads to the definition of five sets of genes defining the five pathways: hypoxia, lactosis, acidosis, lactic acidosis, and lactic acidosis + hypoxia. Of these, we focus on lactic acidosis, hypoxia and acidosis because of their ability to predict outcomes consistently across all five cancer data sets.

The level of expression of a particular pathway within a tumor cell – the "signature" of that pathway in the cell – is calculated on the identified pathway gene sets with some further filtering. The latter simply removes genes that show lower levels of posterior mean expression (< 6 on the RMA scale in both experimental and control group, or < 6 on the RMA scale in the set of five cancer samples). The main goal and effect of this additional filtering is to reduce the pathway gene set sizes.

After obtaining the list of genes to be included in a pathway signature, we examined the first two principal components of expression on these genes across the samples in our experiments. Figures 1 (a), (b) and (c) show the projection of the samples from our experiments onto these two principal components. The projected signatures evaluated on the corrected breast tumor data set from Miller et al. study are also shown, overlaid. These are computed simply by evaluating the linear combination of gene expression values defined by the principal components made on the experimental data. Noting that the second principal components separate the lactic acidosis group from the rest and the hypoxia group from the rest, we use the values on these components to explore in connection with survival outcomes in the breast studies. In the case of acidosis, we use the first principal component of expression of the acidosis genes to score the tumors because this is the component that separates acidosis from the control and lactosis groups.

Evaluation of signatures in breast cancer data sets

Of the five cancer gene expression data sets utilised in this work, the raw data for four were available at the Gene Expression Omnibus (GEO) web site in terms of Affymetrix GeneChip cel

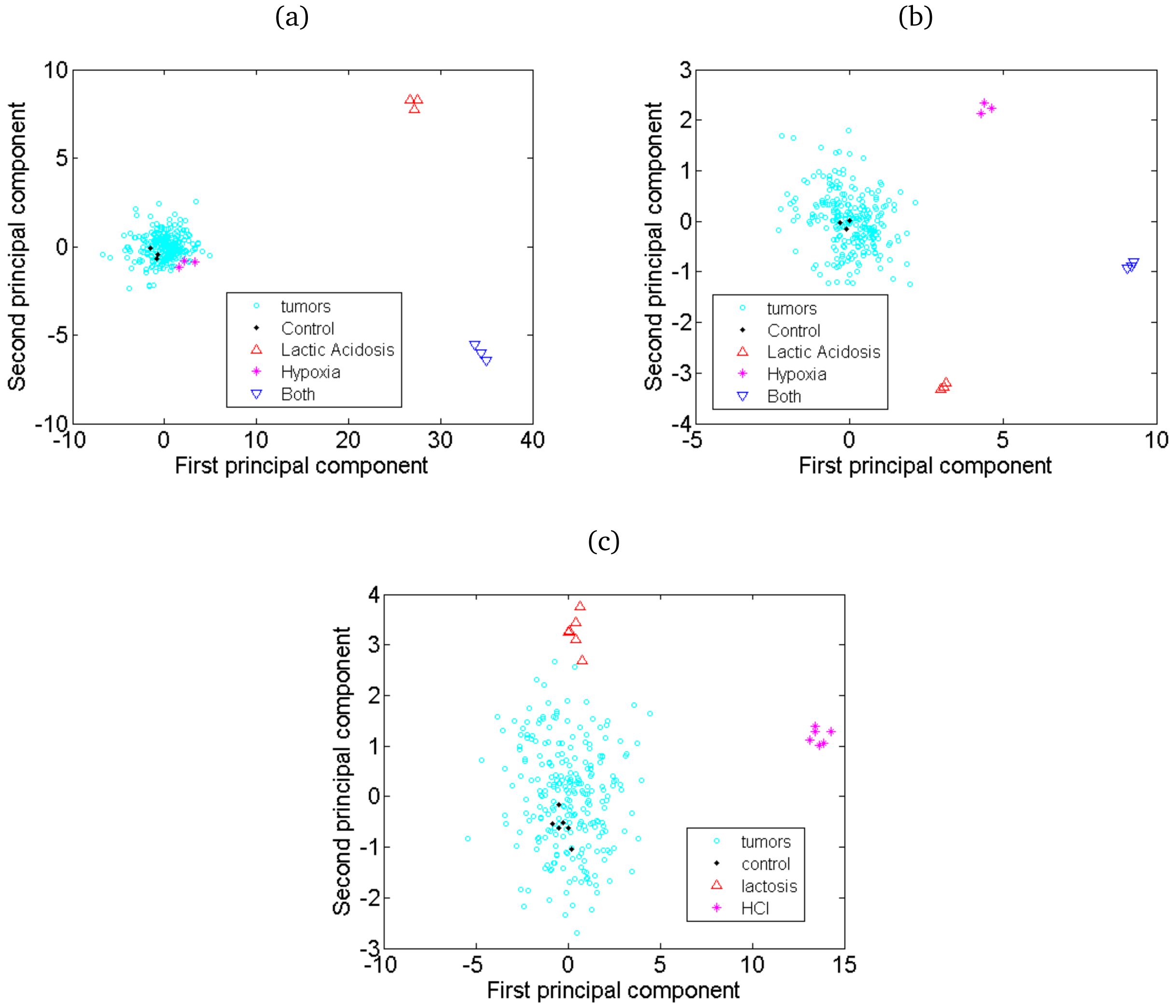

Figure 1: Figures (a), (b) and (c) show scatterplots of the projection of our experimental samples onto the first two principal components of the lactic acidosis, hypoxia and acidosis pathways respectively.

files. Each of these four data sets was separately normalized using standard RMA. The cel files for the fifth data set, that reported in Wang et al., 2005, were not available, but we were able to obtain MAS5.0 data from the authors. We note that this should be borne in mind when considering the analysis results - i.e., that there may be differences in the Wang data analysis that are due primarily or in part to the less robust and less accurate MAS5.0 expression estimates.

For the Sotiriou and Pawitan data sets, we used the clinical data associated with disease free survival rather than time to death to maintain consistency with the Wang data set, and because cause of death is often not due to breast cancer. The clinical data available on GEO from Sotiriou appears to be somewhat corrupt as there are a number of instances of death occurring before relapse. Also, because there are no cases of patients who died without relapse (as opposed to the Pawitan data set) we believe that the indicator for disease free survival in the Sotiriou data set is one in the case of either tumor recurrence or death.

The Kaplan-Meyer curves were drawn by separating the tumor samples according to the level

of expression of their respective signatures. For a given quantile, q, a particular tumor sample was assigned to the high or low expression group depending on whether the score for that tumor was within the qth quantile for all samples in that experiment. The values for q were chosen independently for each tumor data set to maximize the difference between the two curves (irrespective of which group showed better survival). Table 1 lists the quantiles chosen to split the tumor samples for each of the five data sets. Notice that there is surprising consistency among the quantiles associated with a specific signature. This suggests, for example, that among primary breast tumors, approximately 25% will have active lactic acidosis pathways. The same holds true for the acidosis pathway, while there is a much higher probability of an active hypoxia pathway.

Data Set	Lactic Acidosis	Hypoxia	Acidosis
Miller	.21	.65	.20
Wang	.24	.72	.34
Sotiriou	.39	.59	.20
Pawitan	.33	.59	.30
Massague	.25	.52	.24

Table 1: The quantiles that best separate high and low risk patients with primary breast cancer for the five data sets.

In order to ascertain the relationship between our expressoin signatures and known clinical risk predictors such as age of diagnosis, ER status, tumor size, lymph node involvement and Her2 status, we explored survival regression models using the Shotgun Stochastic Search (SSS) framework and software of [4]. The SSS framework is simply a regression model and allows for the evaluation of many combinations of potential predictors (covariates) in the regression. The "search" element refers to computational search over many possible subsets of covariates, and each candidate subset is scored in terms of a relative probability on that model. Other applications of SSS have utilised binary and linear regressions in cancer genomics and molecular phenotyping, as described in studies in [5, 6, 7], for example. Here, the model is a Weibull survival regression model, and we consider as potential covariates all the clinical markers available together with the projected stress response gene expression values on each tumor.

The Wang data set contains only ER status as a clinical variable, and this is a poor predictor for this data set. Clinical predictors for the Pawitan data set were not available. For these reasons, we focused only on the other three data sets for this part of the analysis.

Among the highly scoring survival regression models for the Massague data set, the covariates in the three scoring most highly are: age at diagnosis, and the signature for lactic acidosis + hypoxia (both together, then each as the sole predictor). This combined signature is problematic, as it appears to be a predictor of high likelihood of metastasis (Massague data set) while at the same time a predictor of longer survival in the Wang and Pawitan data sets. The Massague data set is the smallest (containing only 82 samples with survival information). This explains why the fourth best model for the Massague data is the null model.

The Miller data set contains clinical information on ER status, lymph node involvement, and tumor size. All of the models found by SSS include lymph node involvement and tumor size as predictors. The next best predictor is the lactic acidosis signature, which is in two thirds of the models. We find from studying pairwise variable inclusion probabilities that there is generally no replacement of clinical variables by pathway variables. Thus we interpret the large improvements in fitting survival as value added by the signatures.

The Sotiriou data set contains age at diagnosis, ER status, lymph node involvement, and tumor

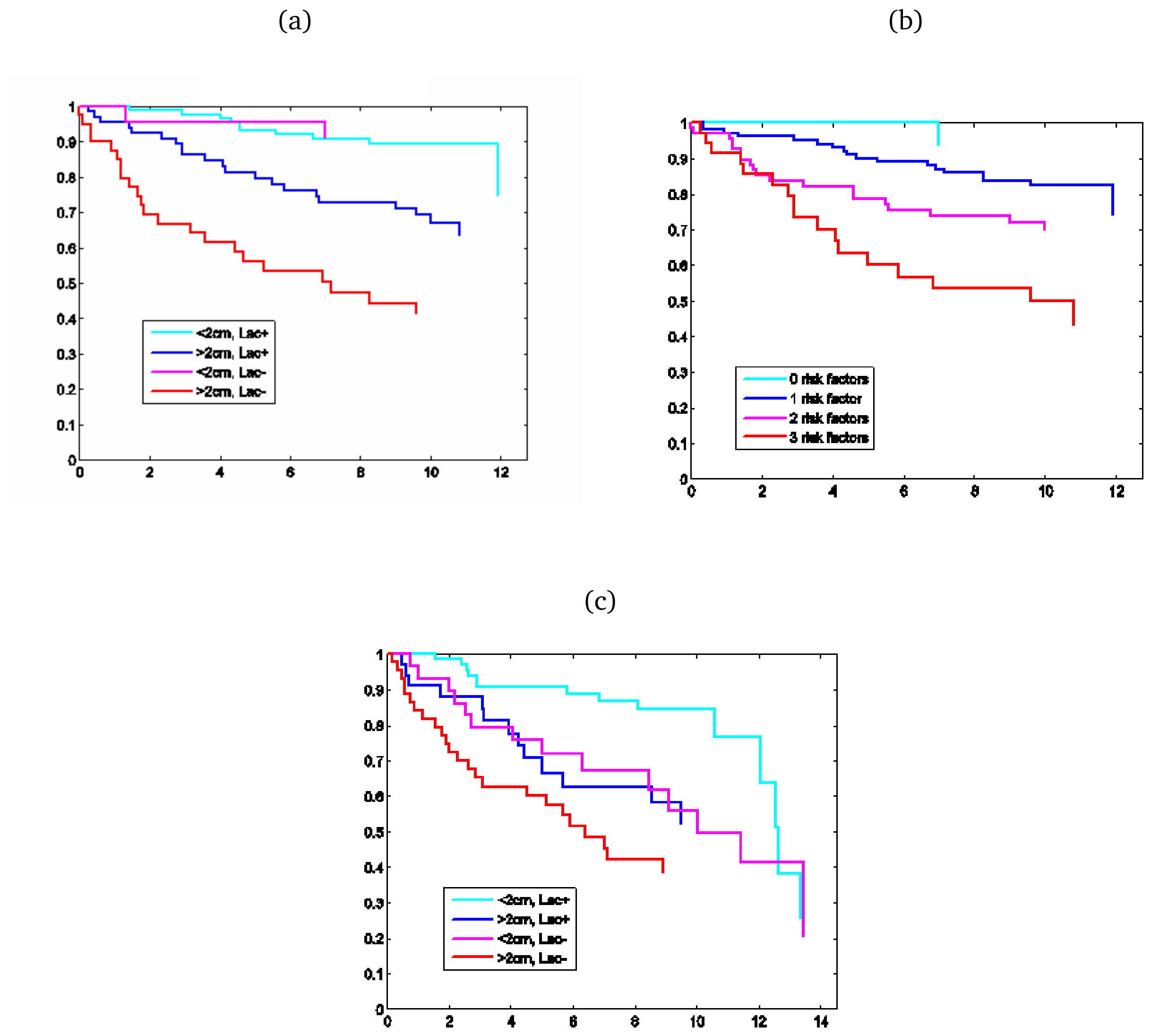

Figure 2: Disease free survival curves for the Miller data set. Figure (a) shows survival when both tumor size and lactic acidosis status are taken into account. For figure (b), the risk factors are 1: tumor size $>$ 2cm, 2: involvement of the lymph nodes, 3: inactive lactic acidosis pathway. Frame (c) is a complementary figure to (a) but now using the Sotiriou data set. Note that the indicator of disease free survival in this data set is 1 for either recurrence or death. Because of this, it is likely that the drop off in disease free survival among the lowest risk group is due to death from causes other than breast cancer.

size. The best clinical predictors in this data set are tumor size and age. The top eight models from SSS for this data set include tumor size, age at diagnosis, lactic acidosis score and hypoxia score in various combinations. Figure 2 (c) is comparable to Figure 2 (a), and again shows synergistic predictive ability between clinical variables and the lactic acidosis signature. Again there is little substitution of pathway variables for clinical variables.

References

[1] Carvalho, C. and Chang, J. and Lucas, J. and Nevins, J. R. and Wang, Q. and West, M. (2008). High-Dimensional sparse factor modelling: Applications in gene expression genomics. *Journal of the American Statistical Association*, (in press). Software: *http://xpress.isds.duke.edu:8080/bfrm/*

[2] J. Lucas, C. Carvalho, Q. Wang, A. Bild, J.R. Nevins and M. West (2006). Sparse statistical modelling in gene expression genomics. In *Bayesian Inference for Gene Expression and Proteomics* (eds. K. Do et al), Cambridge University Press, 155-176.

[3] D.M. Seo, P.J. Goldschmidt-Clermont and M. West (2007). Of mice and men: Sparse statistical modelling in cardiovascular genomics. *Annals of Applied Statistics*, **1**, 152-178.

[4] C. Hans, A. Dobra and M. West (2007). Shotgun stochastic search in regression with many predictors. *Journal of the American Statistical Association*, **102**, 507-516. Software: *http://xpress.isds.duke.edu:8080/sss/*

[5] C. Hans and M. West (2006). High-dimensional regression in cancer genomics. *Bulletin of the International Society for Bayesian Analysis*, **13**, 2-3.

[6] J. Rich, B. Jones, C. Hans, E. Iversen, R. McClendon, A. Rasheed, D. Bigner, A. Dobra, H. Dressman, J. Nevins and M. West (2005) Gene expression profiling and genetic markers in glioblastoma survival *Cancer Research*, **65**, 4051-4058.

[7] H. Dressman, C. Hans, A. Bild, J. Olsen, E. Rosen, P.K. Marcom, V. Liotcheva, E. Jones, Z. Vujaskovic, J. Marks, M.W. Dewhirst, M. West, J.R. Nevins and K. Blackwell (2006) Gene expression profiles of multiple breast cancer phenotypes and response to neoadjuvant therapy *Clinical Cancer Research*, **12**, 819-216.
